# A Series of Novel Pentagonal-Bipyramidal Erbium(III) Complexes with Acyclic Chelating *N_3_O_2_* Schiff-Base Ligands: Synthesis, Structure, and Magnetism

**DOI:** 10.3390/molecules26226908

**Published:** 2021-11-16

**Authors:** Tamara A. Bazhenova, Vyacheslav A. Kopotkov, Denis V. Korchagin, Yuriy V. Manakin, Leokadiya V. Zorina, Sergey V. Simonov, Ilya A. Yakushev, Vladimir S. Mironov, Alexander N. Vasiliev, Olga V. Maximova, Eduard B. Yagubskii

**Affiliations:** 1 Institute of Problems of Chemical Physics, IPCP RAS, Chernogolovka 142432, Russia; tabazh@mail.ru (T.A.B.); korden@icp.ac.ru (D.V.K.); george88@bk.ru (Y.V.M.); zorina@issp.ac.ru (L.V.Z.); simonovsv@rambler.ru (S.V.S.); cs68@mail.ru (I.A.Y.); mirsa@list.ru (V.S.M.); yagubski@gmail.com (E.B.Y.); 2 Institute of Solid State Physics, ISSP RAS, Chernogolovka 142432, Russia; 3 Kurnakov Institute of General and Inorganic Chemistry, IGIC RAS, Moscow 119333, Russia; 4 Shubnikov Institute of Crystallography of Federal Scientific Research Centre “Crystallography and Photonics” RAS, Moscow 119333, Russia; 5 Laboratory of Quantum Functional Materials, National University of Science and Technology “MISiS”, Moscow 119049, Russia; anvas2000@yahoo.com; 6 Lomonosov Moscow State University, Moscow 119991, Russia; olga.reukova@mail.ru

**Keywords:** field-induced single-molecular magnets, seven-coordinate complexes, Er(III) complexes, ligands H_2_DAPMBH and H_4_DAPS, crystal structure, DC and AC magnetic properties

## Abstract

A series of six seven-coordinate pentagonal-bipyramidal (PBP) erbium complexes, with acyclic pentadentate [*N_3_O_2_*] Schiff-base ligands, 2,6-diacetylpyridine bis-(4-methoxybenzoylhydrazone) [H_2_DAPMBH], or 2,6-diacethylpyridine bis(salicylhydrazone) [H_4_DAPS], and various apical ligands in different charge states were synthesized: [Er(DAPMBH)(C_2_H_5_OH)Cl] (**1**); [Er(DAPMBH)(H_2_O)Cl]·2C_2_H_5_OH (**2**); [Er(DAPMBH)(CH_3_OH)Cl] (**3**); [Er(DAPMBH)(CH_3_OH)(N_3_)] (**4**); [(Et_3_H)N]^+^[Er(H_2_DAPS)Cl_2_]^−^ (**5**); and [(Et_3_H)N]^+^[Y_0_._95_Er_0_._05_(H_2_DAPS)Cl_2_]^−^ (**6**). The physicochemical properties, crystal structures, and the DC and AC magnetic properties of **1**–**6** were studied. The AC magnetic measurements revealed that most of Compounds **1**–**6** are field-induced single-molecule magnets, with estimated magnetization energy barriers, *U*_eff_ ≈ 16–28 K. The experimental study of the magnetic properties was complemented by theoretical analysis based on ab initio and crystal field calculations. An experimental and theoretical study of the magnetism of **1**–**6** shows the subtle impact of the type and charge state of the axial ligands on the SMM properties of these complexes.

## 1. Introduction

**Scheme 1:**
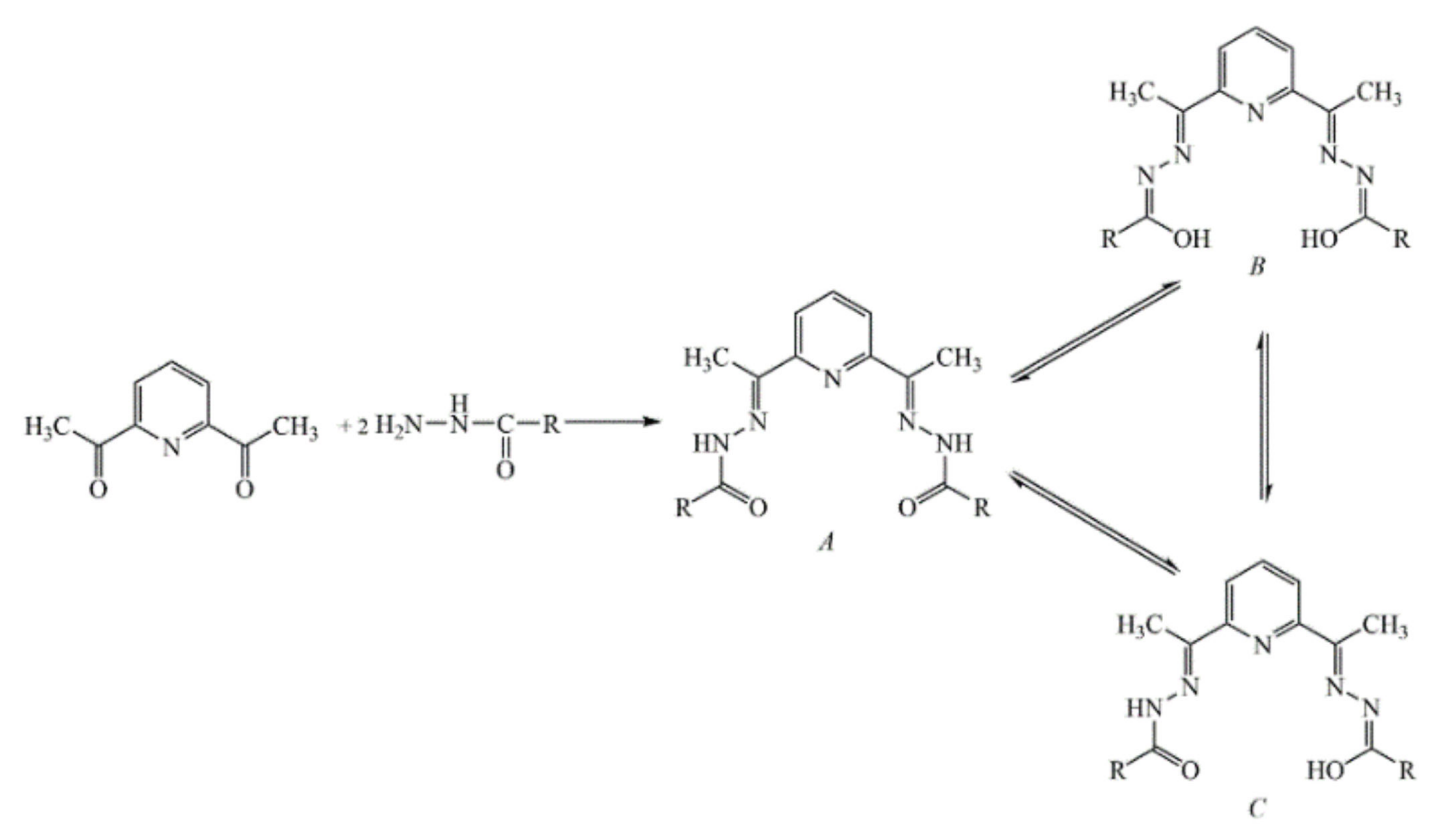
Diacetylpyridine-based acyclic pentadentate (*N_3_O_2_*) ligands and their possible isomeric forms (A, B, C): R = 2-OHC_6_H_4_(H_4_DAPS); 4-OCH_3_C_6_H_4_(H_2_DAPMBH); and C_6_H_5_ (phenyl, H_2_DAPBH).

Currently, it has become obvious that magnetic anisotropy is the most critical factor for the development of high-performance single-molecule magnets (SMMs) [1,2,3]. An effective strategy for increasing the magnetization reversal barriers (*U*_eff_) and the blocking temperature (*T*_B_) of molecular nanomagnets is based on the use of coordination metal centers with enhanced spin–orbit coupling (provided by the 4*d*, 5*d*, and 4*f* elements) [4,5,6,7,8], which are capable of producing strong uniaxial magnetic anisotropy, especially in the case of a suitable coordination environment [6,9,10,11,12,13,14,15]. An alternative way to achieve high SMM characteristics is to control the coordination geometry and the symmetry around the lanthanide ions with the aim of quenching the unwanted quantum tunneling of magnetization (QTM) through the use of appropriately designed molecules with higher-order symmetries [3]. These factors, taken together, ensure the unquenched angular orbital momentum of the metal center, resulting in strong uniaxial magnetic anisotropy. The geometry of the ligand environment can enhance the local magnetic anisotropy [9,12,13,14,15]. Experimental and theoretical studies of seven-coordinated metal centers with pentagonal-bipyramidal (PBP) geometry (with a pseudo *D*_5h_ symmetry) show that such centers are promising as anisotropic spin carriers [9,11,16,17,18,19,20,21,22,23,24]. As a result, new PBP complexes of 3*d*, 4*d*, 5*d* and 4*f* metals have been synthesized in recent years [25,26,27,28,29,30,31,32,33,34]. Among them, of special note are the seven-coordination PBP Dy complexes displaying high *U_eff_* and *T_B_* values, up to 1800 K and 20 K, respectively [3,19,30,31,32,33,34]. These complexes contain weak donor ligands in the equatorial plane, particularly five water or pyridine molecules, and strong bulky ligands in the axial positions, such as tricyclohexylphosphine oxide or *tert*-butoxide. For the Dy^3+^ ions with an oblate 4*f* electron density, such a coordination environment provides a very strong axial and weak equatorial crystal field (CF), resulting in high values of *U_eff_* and *T_B_* [24,35,36,37]. However, the PBP geometry in these complexes is largely random, since the Ln^3+^ ions prefer large coordination numbers (8–10) and variable coordination geometry because of their large ionic size and highly shielded core-like 4*f* orbitals. At the same time, there is a family of acyclic pentadentate (*N_3_O_2_*) ligands obtained from the condensation reactions of 2,6-diacetylpyridine with various hydrazides (Scheme 1), which are widely used for the targeted synthesis of the PBP complexes of 3*d* metals. In these complexes, depending on the synthesis conditions, the ligand forms a rigid neutral or charged (−1 or −2) pentagonal-equatorial plane around the metal ion, and the final coordination of PBP is completed by two apical ligands (usually solvent molecules and/or various simple anions) [38,39,40,41,42,43,44,45,46,47,48,49,50,51,52]. In 2018, Sutter’s group first synthesized the isostructural PBP complexes of Dy and Tb using an acyclic pentadentate ligand, 2,6-diacethylpyridine bis(salicylhydrazone) (H_4_DAPS, Scheme 1): (Et_3_NH)^+^[Ln^III^(H_2_DAPS)Cl_2_]^-^ (Ln= Tb, Dy, Y_0_._94_Dy_0_._06_) [53]. Unlike the Tb complex, the Dy complex is a field-induced single-ion magnet. For a Dy analog containing 95% of diamagnetic Y, the value of the magnetization barrier is 70 K under a low DC field, equal to 500 Oe. Despite the fact that the seven-coordinated PBP Dy complexes are being actively studied [33,34], we are aware of only three works in which the PBP Er complexes with pseudo-*D*_5h_ symmetry have been described [32,54,55]. Considering that Dy and Er have fundamentally different distributions of 4*f* electron density, oblate and prolate, respectively [35], it is of interest to synthesize PBP erbium complexes with acyclic pentadentate (*N_3_O_2_*) ligands and to perform a comparative study of their SMM properties, depending on the nature of the axial ligands. This study is motivated by the fact that the strength of the crystal field of the axial ligands is crucially important for obtaining the strong axiality of the magnetic anisotropy of Ln complexes that minimizes the transverse magnetic anisotropy and suppresses the QTM processes, shortcutting the energy barrier, *U*_eff_; ultimately, this results in high SMM performance [30,31,32,33,34,35].

Herein, we present a novel series of pentagonal-bipyramidal Er complexes with equatorial-pentadentate (*N_3_O_2_*) ligands (Scheme 1) and axial ligands in different charge states: [Er(DAPMBH)(C_2_H_5_OH)Cl] (**1**); [Er(DAPMBH)(H_2_O)Cl] 2C_2_H_5_OH (**2**); [Er(DAPMBH)(CH_3_OH)Cl] (**3**); [Er(DAPMBH)(CH_3_OH)(N_3_)] (**4**); [(Et_3_H)N]^+^[Er(H_2_DAPS)Cl_2_]^−^ (**5**); and [(Et_3_H)N]^+^[Y_0_._95_Er_0_._05_(H_2_DAPS)Cl_2_]^−^ (**6**). The DC and AC magnetic properties were studied and supplemented by a theoretical analysis based on ab initio calculations and the crystal field theory. The influence of the charge state of the axial ligands on the single-molecule magnetic properties is analyzed in terms of our experimental and theoretical results.

## 2. Results and Discussion

### 2.1. Synthesis and Molecular Structure

To study the impact of the charge states of the axial ligands on the magnetic behavior of the Er^+3^ pentagonal-bipyramidal (PBP) complexes in an alternating magnetic field, we synthesized a series of six seven-coordinate PBP complexes, including a plane pentadentate ligand with a [*N_3_O_2_*]^2−^ binding node in the equatorial position, and various axial ligands.

Most of the complexes described in this work, in particular, the neutral complexes, **1**–**4**, were obtained using the pentadentate ligand with the [*N_3_O_2_*]-binding node, 2,6-diacetylpyridine bis-(4-methoxybenzoylhydrazone) [H_2_DAPMBH], that was synthesized by us via a ketone-hydrazine condensation reaction between 1 equivalent of 2,6-diacetylpyridine and 2 equivalents of 4-methoxybenzoylhydrazine, in 96% ethanol [56].

We found that the interaction of anhydrous ErCl_3_ with H_2_DAPMBH in absolute C_2_H_5_OH in the presence of 2 equivalents of the deprotonating agent, Et_3_N, leads to the formation and precipitation of pure neutral complex, [Er(DAPMBH)Cl(C_2_H_5_OH)] (**1**), practically insoluble in absolute ethanol with a very high yield. We failed to obtain the crystals of Complex **1** suitable for X-ray diffraction from ethyl alcohol. Its composition was found from elemental analysis and IR spectra.

**Figure 1:**
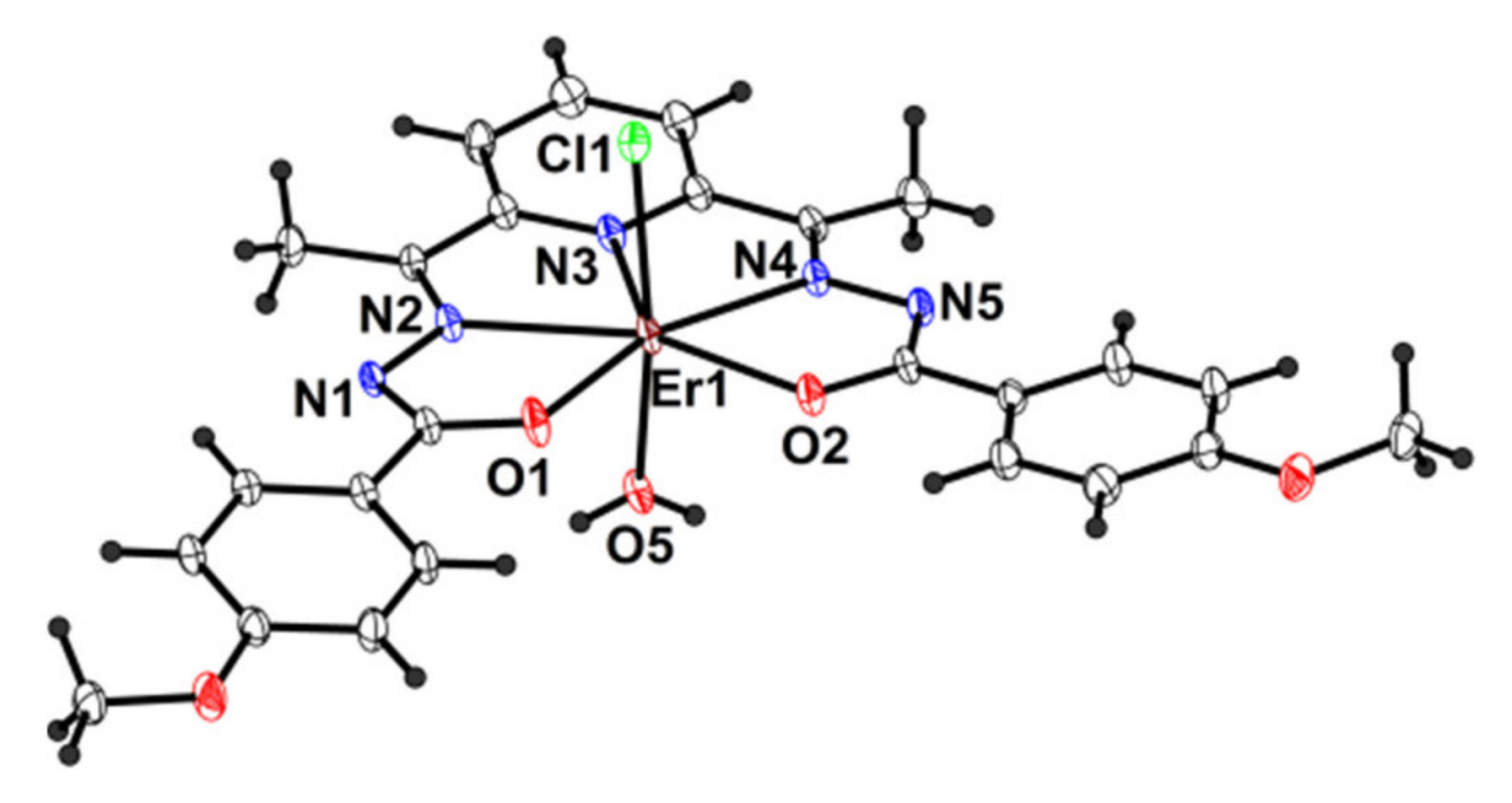
Molecular structure of the neutral complex, [Er(DAPMBH)Cl(H_2_O)], in **2**.

If the reaction is carried out in ethyl alcohol containing up to 5% water and rectified alcohol, the complex, [Er(DAPMBH)Cl(H_2_O)] 2C_2_H_5_OH (**2**), is formed as the main product, as seen in Figure 1.

**Figure 2:**
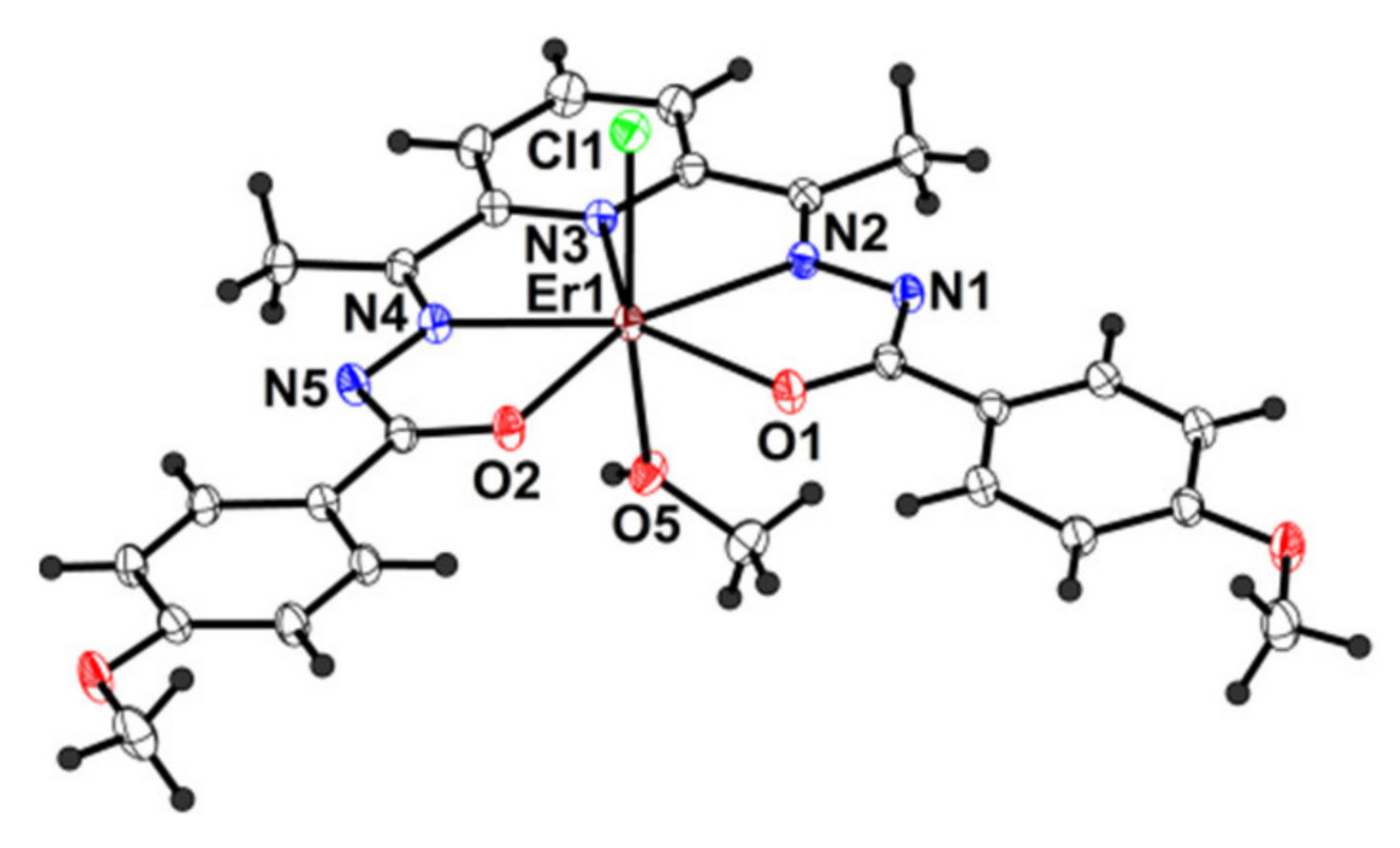
Molecular structure of the neutral complex, [Er(DAPMBH)Cl(CH_3_OH)], in **3**.

Complex **2** is very soluble in aqueous ethanol and precipitates in the form of crystals only with significant evaporation of the solution. Attempts to obtain crystals from **1,** suitable for X-ray structural analysis by the recrystallization of Powder **1** from another solvent, for example, methanol, were also unsuccessful. The erbium-coordinated C_2_H_5_OH molecule in Complex **1** is easily replaced by CH_3_OH when **1** is dissolved in methanol. As a result of this substitution, Complex **3** is formed, as shown in Figure 2.

**Figure 3:**
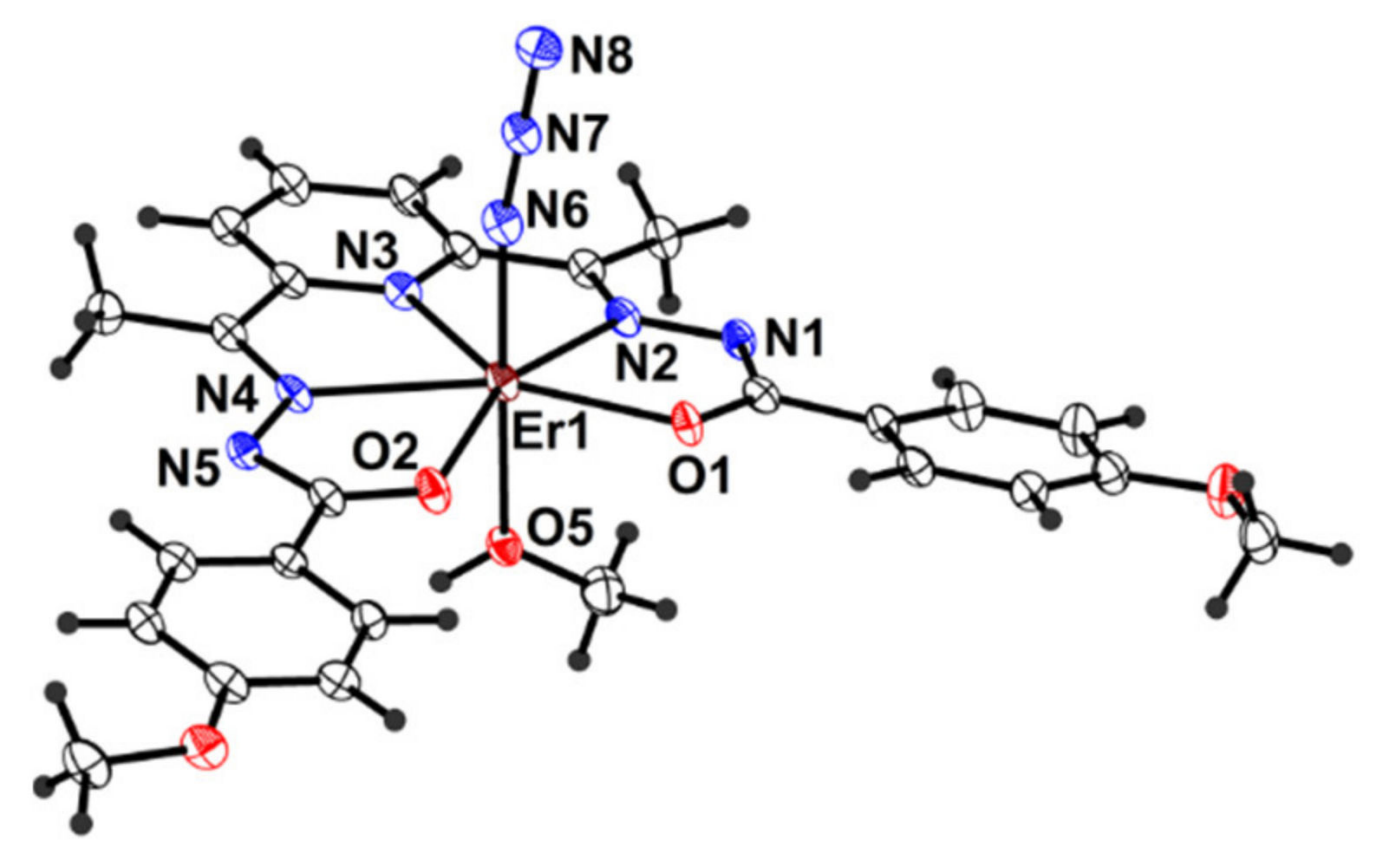
Molecular structure of the neutral complex, [Er(DAPMBH)(CH_3_OH)(N_3_)], in **4**.

Since **3** is very soluble in methanol, a significant concentration of the solution is required to obtain crystals, similar to the case of Complex **2**. Chloride ligand in Complex **3**, in turn, can be replaced by an azide ion with the slight heating of the methanol solution of **3** with an excess of NaN_3_. The reaction results in the formation of a neutral seven-coordination complex of erbium, [Er(DAPMBH)(CH_3_OH)(N_3_)] (**4**), containing a terminal azide ligand, as shown in Figure 3.

Each of these four complexes, **1**–**4**, contains the fully deprotonated ligand [DAPMBH]^2–^ in the equatorial position, and one charged and one neutral ligand in the axial positions.

**Figure 4:**
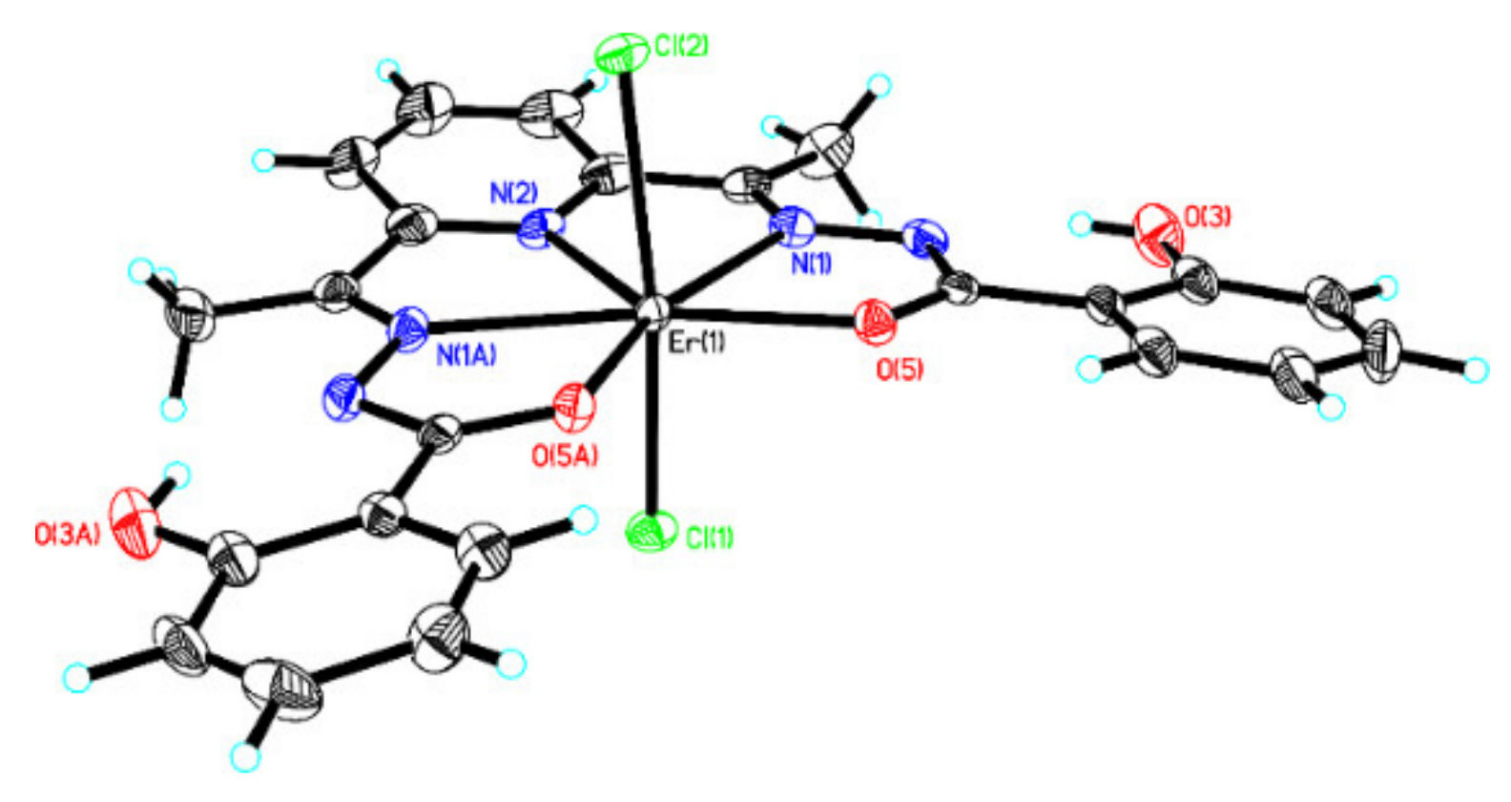
Molecular structure of the anionic complex, [Er(H_2_DAPS)Cl_2_]^−^, in **5**.

Using the ligand, [H_2_DAPMBH], we were unable to isolate the anionic complex, [(Et_3_H)N]^+^[Er(DAPMBH)(Cl_2_)]^−^, with two charged chloride axial ligands. In the context of our study, this complex was originally intended to correctly compare the effect of the charge states of the axial ligands on the magnetic properties in a series of PBP erbium complexes with the same equatorial ligand. The reaction of ErCl_3_ with H_2_DAPMBH has always led to a neutral Complex **1**. The required erbium complex with the [*N_3_O_2_*]^2−^ ligand in the equatorial position and the two charged axial ligands could be isolated only using the related ligand, H_4_DAPS, containing OH groups in the ortho positions of the phenyl rings [43], instead of H_2_DAPMBH. The interaction of ErCl_3_ with H_4_DAPS in absolute ethanol in the presence of the deprotonating agent, Et_3_N, leads, as we have shown, to the desired erbium complex, [(Et_3_H)N]^+^[Er(H_2_DAPS)Cl_2_]^−^ (**5**) (Figure 4). An analogous anionic Er complex was recently synthesized, but with a different counterion, [(CH_3_)_4_N]^+^ arising from the use of the deprotonating agent, (CH_3_)_4_NOH [54].

Note that Compound **5** is an erbium analogue of the dysprosium complex, as has been described in [53]. It is also interesting to note that all attempts to obtain neutral dysprosium complexes similar to the erbium complexes, **1**–**4**, with the ligand, H_2_DAPMBH, were unsuccessful. Under these conditions, for dysprosium, we observed the formation of only ionic complexes of the type [(Et_3_H)N]^+^[Dy(DAPMBH)Cl_2_]^−^, similar to those described in [53].

Thus, all the obtained erbium complexes, **1**–**6**, contain pentadentate ligands with the [*N_3_O_2_*]^2−^ binding node in the equatorial plane and two axial ligands represented by both negatively charged groups (N_3_^−^, Cl^−^), and neutral molecules (C_2_H_5_OH, CH_3_OH, H_2_O). The shape analysis of these seven-coordinate compounds reveals a distorted PBP geometry with a D*_5h_* (pseudo) CF symmetry around the Er ions (Table S1, Supplementary Materials).

### 2.2. Description of the Structure

Complexes **2** and **3** crystallize in the triclinic space group, *P*$\bar{1}$, with one formula unit per asymmetric unit, and all components in general positions (Figures S1 and S3). The structure of **2** comprises ethanol solvent molecules that show positional disorder. Compound **4** crystallizes in the monoclinic space group, *P*2_1_/*c*, and an asymmetric unit contains one Er complex in a general position (Figure S5). Compounds **5** and **6** crystallize in the orthorhombic space group, *Cmc*2_1_, with half of the formula unit per asymmetric unit; both the cation and anion are located in partial positions on a mirror plane, and one ethylene group of the cation is disordered about this plane (Figure S9). Complexes **5** and **6** are isostructural to each other and to the previously reported Dy and Tb analogues [53]. The key bond distances and angles in the structures investigated are listed in Table S2 (for **2**),  Table S4 (**3**), S6 (**4**), and S8 (**5** and **6**).

The molecular structures of the complexes are shown in Figure 1, Figure 2, Figure 3 and Figure 4. In all the structures, the central 4*f*-metal (Er, or Y in **6**) is seven-coordinated by three N and two O atoms in the equatorial plane of the chelating pentadentate ligand, and two atoms (Cl, and N or O) of the axial ligands, Clˉ, N_3_ˉ, and H_2_O, CH_3_OH or C_2_H_5_OH. The DAPMBH/H_2_DAPS ligand is deprotonated on both N sites and has a two-charge state. The Er(III) complexes are neutral if one of the axial ligands is negatively charged (**2**–**4**), or anionic if they contain two negatively charged Clˉ axial ligands and a (Et_3_H)N^+^ counterion (**5**, **6**). There are no noticeable differences in the bond length values in the Er polyhedrons for complexes with different charges (Table S9). The Er-O_equatorial_ and Er-N_equatorial_ bond lengths to the pentadentate ligand in **2**–**6** are in the range of 2.22–2.28 Å and 2.40–2.45 Å, respectively. The Er-O_axial_ bond lengths to the axial ligands in the neutral complexes, **2**–**4**, with H_2_O/CH_3_OH ligands, range between 2.27 and 2.33 Å. The Er-N_axial_ and Er-Cl_axial_ bond lengths are 2.23 Å and 2.59–2.66 Å, respectively.

The DAPMBH and H_2_DAPS ligands are not quite planar: the dihedral angle between the two semicarbazone planes of the ligand, defined by seven nonmetallic atoms of two pentagonal cycles (for example, O1, C8, N1, N2, C10, C11, N3, and O2, C18, N5, N4, C16, C15, N3 in Figure S1) is 4.02(8)° in **2**, 6.74(5)° in **3**, and 6.0(2)° in **4**. The same angle in the H_2_DAPS ligand is 5.3(2)° in **5,** and 5.4(1)° in **6**.

In all structures, the metal complexes are well-isolated molecular units. Their contacts with neighboring complexes, and with incorporated counterions or solvent molecules, are mediated by hydrogen bonding and/or π-stacking [57,58,59] (Figures S2, S4, S6–S8, S10 and S11).

**Figure 5:**
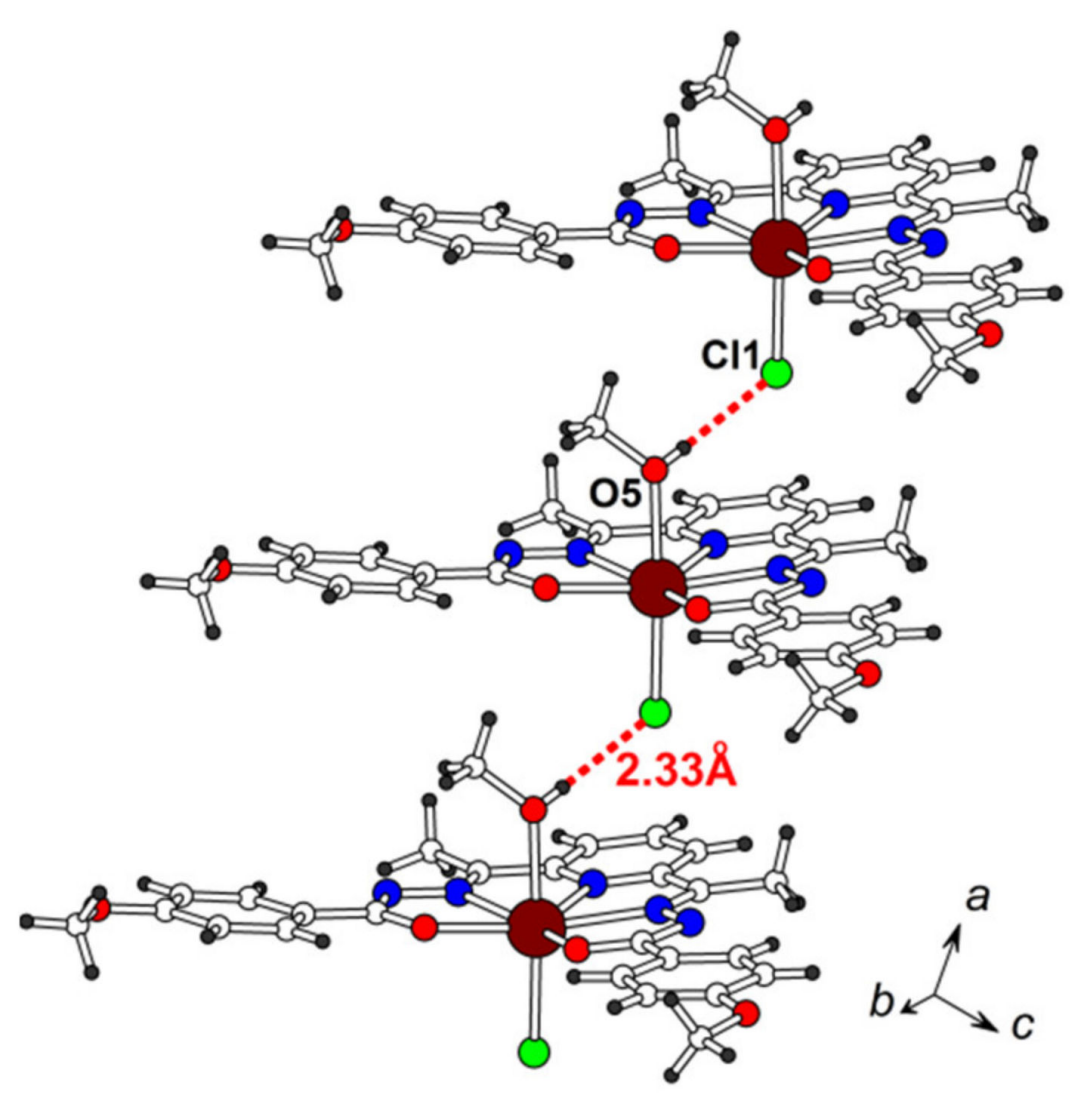
Infinite chain of hydrogen-bonded Er complexes in **3**. Er…Er intrachain distance is 7.0338(2) Å.

The hydrogen bonding can consolidate the adjacent metal complexes into dimeric or chain assemblies. H-bonded infinite chains of the Er complexes are found in the structure of **3**. Along the chain, O-H…Cl bridges (with a H…Cl distance of 2.33 Å) between the MeOH and Cl ligands of the adjacent Er units are formed, and the Er…Er intrachain distance is 7.0338(2) Å (Figure 5). π-stacking is observed only between the chains (Figure S4b). 

**Figure 6:**
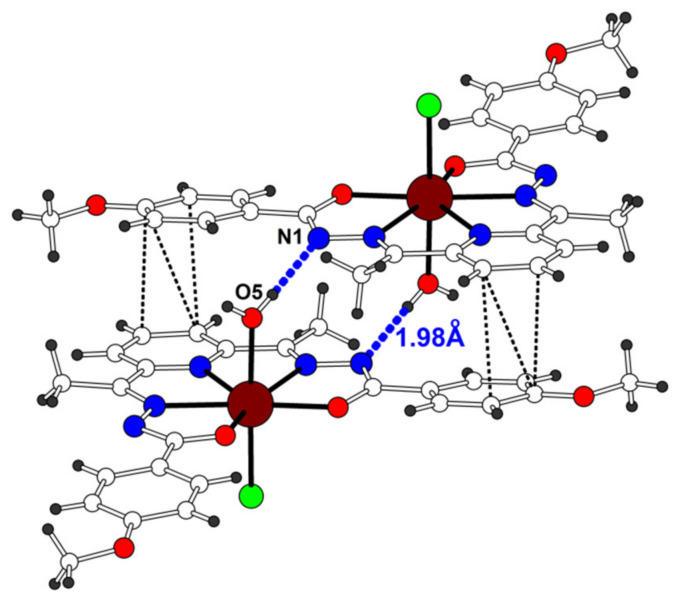
Centrosymmetric H-bonded dimers in **2**. C-H…N bonds are shown by blue dashed lines. C…C contacts < 3.6 Å are shown by black dotted lines. The Er…Er intradimer distance is 7.0386(4) Å in **2**.

Hydrogen-bonded centrosymmetric dimers are found in the structures of **2** and **4**. In **2**, two Er complexes are connected through a pair of equivalent hydrogen O(5)_H2O_-H…N(1)_DAPMBH_ bonds (H…N of 1.98 Å), with an Er…Er intradimer separation of 7.0386(4) Å (Figure S2 and Figure 6). The π-π interactions are observed both inside and between the dimers (C…C intradimer distances less than sum of van der Waals radii are marked by black dotted lines in Figure 6). In **4**, the molecules are paired via two equivalent hydrogen bonds, O(5)_H2O_-H…N(5)_DAPMBH_ (Figure S6), similar to those in **2**, but with a considerably shorter H…N bond distance (1.80 Å). This also leads to a short Er…Er intradimer separation of 6.6939(17) Å, the smallest among the considered structures. In addition, a number of short π-π contacts are observed inside the dimer and between these units in the crystal structure packing (details are provided in the Supplementary Section).

In the other compounds (**5**,**6**), the neighboring metal complexes are less connected to each other. Crystal packing diagrams show that the shortest intermolecular Er−Er distance in the structure of **5** is ∼7.6 Å (Figure S10). As previously noted for the Dy and Tb analogues of Complexes **5** and **6** [53], there is essentially no short intermolecular contacts in the crystal structure, which could cause a magnetic superexchange pathway. Indeed, a more detailed analysis of the crystal structure of **5** reveals only weak C-H...Cl(2) (H...Cl of 2.75 Å) van der Waals interactions between the anionic complexes [Er(H_2_DAPS)Cl_2_]^−^, while the intermolecular hydrogen bond, Cl(2)…H-N (Cl…H of 2.19 Å), between the anionic complex and the cation [(Et_3_H)N]^+^ is observed, as shown in Figure S11. The Supplementary section contains more information about molecular packing in the structures of **2**–**6**.

### 2.3. Magnetic Properties

#### 2.3.1. Static (DC) Magnetic Properties

**Figure 7:**
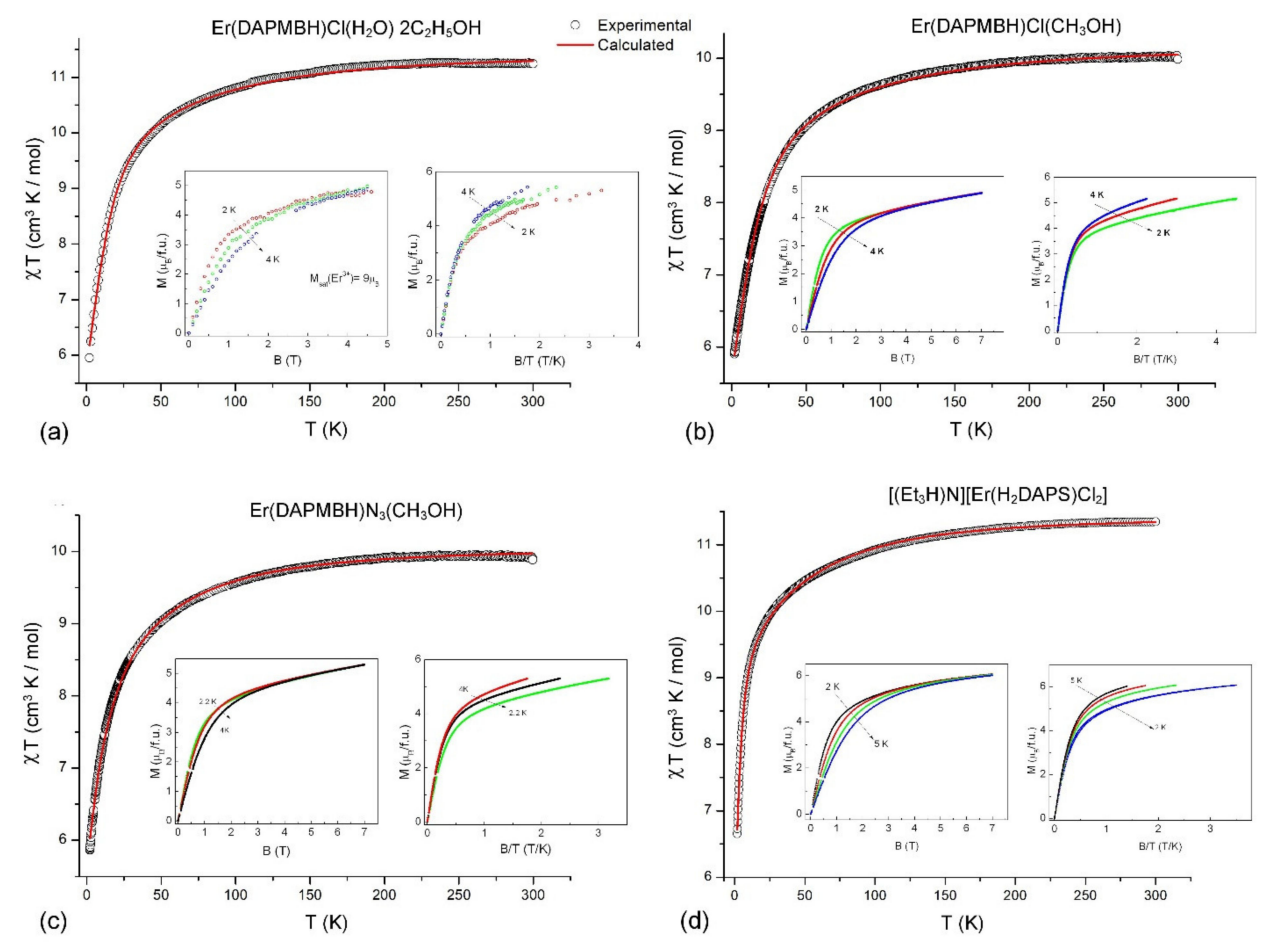
Experimental (open circles) and calculated (solid red lines) temperature dependences of magnetic susceptibility (in the form of *χT* vs. *T*) of (**a**) **2**, (**b**) **3**, (**c**) **4**, and (**d**) **5**. In the insets: field dependence of magnetization plotted in *M* vs. *B* (left panels), and the *M* vs. *B/T* plot at different temperatures (right panels).

The temperature dependences of the magnetic susceptibility for Complexes **2**–**5** were measured in the temperature range of 2–300 K, in the field-cooled (*FC*) mode, at a 1000 Oe DC magnetic field, as shown in Figure 7. At room temperature, the *χ*_mol_*T* product of **2** and **5** is close to the free-ion value of Er^3+^, 11.48 cm^3^ K mol^−1^; in Compounds **3** and **4**, *χ*_mol_*T* is somewhat lower, likely because of the reduced concentration of Er^3+^ ions in the powder samples. Upon cooling from room temperature, the *χ*_mol_*T* product of **2**–**5** gradually decreases and then drops to c.a. 6 cm^3^ K mol^−1^ below 100 K because of the thermal depopulation of the exited Stark levels of the Er^3+^ ion. The field dependencies of the magnetization (*M*/µ_B_ vs. *B/T*) for all the complexes have been measured at temperatures of 2 K–5 K in the field range of 0–7 T (Figure 7 (left panels)). The magnetization of **2**–**5** does not saturate and reaches the values of 4.85 (5 T), 4.88 (7 T), 5.3 (7 T), and 6.01 (7 T) µ_B_, respectively, at 2 K. The magnetic field dependences of magnetization, plotted on the *M* vs. *H/T* axes at different temperatures, do not coincide (Figure 7 (right panels)), signifying the considerable single-ion magnetic anisotropy of these complexes.

#### 2.3.2. Crystal Field Analysis

To obtain more insight into the magnetic properties of Complexes **2**–**5**, we performed a crystal field (*CF*) analysis of the Er^3+^ ion. To this end, we simulated the DC magnetic properties (see Figure 7) using the CF theory for lanthanide ions, which is based on the CF Hamiltonian *H*, composed of the free-ion part, *H*_0_, and the CF term, *H*_CF_:

(1) $$H=H_{0}+H_{\mathrm{CF}},$$

The structure of the free-ion Hamiltonian *H*_0_ is given by:

(2) $$H_{0}=\underset{k=2,4,6}{\sum }f_{k}F^{k}+\zeta_{4f}\underset{i}{\sum }l_{i}s_{i}+\alpha L\left(L+1\right)+\beta G\left(R_{7}\right)+\gamma G\left(G_{2}\right),$$

where *f_k_* and *F^k^* are the angular and radial Slater parameters, respectively; the second term is the spin-orbit operator; and *α*, *β*, and *γ* are the two-particle configuration interaction parameters (also known as the Trees parameters) [60,61,62]. The *H*_CF_ Hamiltonian describes the metal–ligand interactions in the frame of the Wybourne CF formalism:

(3) $$H_{CF}=\underset{k,q}{\sum }B_{kq}C_{q}^{k},$$

where *B_kq_* are the CF parameters, (*k* = 2,4,6; *q* ≤ *k*); and *C_q_^k^* are the spherical tensor operators for the *f*-electrons [60,61,62]. The *B_kq_* parameters are adjustable parameters, which are usually obtained from the simulation of the optical or magnetic data for the lanthanide compounds. Numerous examples of CF calculations for Ln^3+^ ions have been extensively reported in the literature [60,61,62]. In our work, the CF calculations are based on the simulation of the DC magnetic susceptibility of the lanthanide complexes, **2**–**5** (Figure 7). In magnetically anisotropic lanthanide systems, the magnetization, ***M***, and the applied magnetic field, ***H***, are not necessarily collinear; they are related by the tensor, ***χ***, of the anisotropic magnetic susceptibility, ***M*** = ***χH***. In a coordinate system (*x*, *y*, *z*), ***χ*** is represented by a 3 × 3 matrix, *χ_αβ:_*

(4) $$M_{\alpha }=\underset{\beta }{\sum }\chi_{\alpha \beta }H_{\beta }\;,\;$$

where *α*, *β* = *x*, *y,* or *z*. The components, *χ_αβ_*, of the ***χ*** tensor are calculated in terms of the eigenvectors, |*i*>, and the energies, *E_i_*, of the CF Hamiltonian (1), using the Gerloch–McMeeking equation [63]:

(5) $$\chi_{\alpha \beta }=\frac{N_{a}}{\sum_{i}\exp\;(-E_{i}/kT)}\underset{i}{\sum }\left\{\underset{j}{\sum }\frac{\langle i\left|\mu_{\alpha }\right|j\rangle \langle j\left|\mu_{\beta }\right|i\rangle }{kT}-\underset{j\neq i}{\sum }\frac{\langle i\left|\mu_{\alpha }\right|j\rangle \langle j\left|\mu_{\beta }\right|i\rangle +\langle i\left|\mu_{\beta }\right|j\rangle \langle j\left|\mu_{\alpha }\right|i\rangle }{E_{i}-E_{j}}\right\}\exp\;(-E_{i}/kT)$$

where *N_a_* is the Avogadro number; *E_i_* is the energy of the CF state; |*i*>, *k* is the Boltzmann constant; *T* is the absolute temperature; and *µ_α_*, *µ_β_* are the components of the operator of the magnetic momentum:

(6) $$\mu =-\mu_{B}\left(L+2S\right)\;,$$

where ***L*** and ***S*** are, respectively, the operators of the total orbital momentum and spin, and *µ*_B_ is the Bohr magneton. The eigen values of the 3 × 3 matrix *χ_αβ_* (5) are the principal components of the anisotropic magnetic susceptibility (*χ_x_*, *χ_y_* and *χ_z_*); the powder magnetic susceptibility is their average value, *χ* = (*χ_x_* +*χ_y_* +*χ_z_*)/3.

With this background, the energies of the CF states that *E_i_*, and their wave functions, |*i*>, can be obtained from the simulation of the DC magnetic susceptibility of **2**–**5**, in terms of Equations (1)–(6). The CF parameters, *B_kq_*, are obtained from the fitting of the simulated *χT* curve to the experimental DC magnetic data (see (Figure 7). However, for the low-symmetry lanthanide complexes, **2**–**5**, these CF calculations are problematic because of the strong overparametrization of the fitting to the *χT* curves, which involves 27 variable *B_kq_* parameters for the *C*_1_ point symmetry of the Er^3+^ ions in **2**–**5**. To reduce the number of variables, we used the superposition CF model [64,65,66], which relates the *B_kq_* parameters with the geometry of the metal site in terms of the intrinsic CF parameters, *b_k_*(*R*_0_), referring to the local metal–ligand interactions:

(7) $$B_{kq}=\underset{n}{\sum }b_{k}\left(R_{0}\right){\left(R_{0}/R_{n}\right)}^{t_{k}}C_{q}^{k}\left(\theta_{n},\varphi_{n}\right),$$

where the index *n* runs over the metal–ligand pairs in the coordination polyhedron of the Ln^3+^ ion; *b_k_*(*R*_0_) are the three (*k* = 2,4,6) intrinsic CF parameters; (*R_n_*, *θ_n_*, *φ_n_*) are the polar coordinates of the *n*-th ligand atom; *t^k^* are the power-law exponents; and *R*_0_ is the reference metal–ligand distance. More information on the superposition of the CF model, and its applications for Ln compounds, can be found in the literature [64,65,66].

The *B_kq_* parameters are obtained from the best fit to the experimental *χT* curves of **2**–**5** (Figure 7). In this computational scheme, the intrinsic CF parameters *b_k_*(*R*_0_) vary independently for the O, N, and Cl coordinating atoms. For each of them, the reference distances, *R*_0_, are set to the average metal–ligand distances, (*R*_0_(O) = 2.25 Å, *R*_0_(N) = 2.42 Å, and *R*_0_(Cl) = 2.60 Å), and the power-law indexes, *t*^k^, in (7) are fixed at *t*^2^ = 5, *t*^4^ = 8, and *t*^6^ = 11 [64,65,66]. The polar coordinates (*R_n_*, *θ_n_*, *φ_n_*) in (7) describe the atomic positions of the O, N, and Cl atoms of the coordination polyhedra in **2**–**5**. The atomic parameters (*F*^2^, *F*^4^, *F*^6^, *ξ*_4f_, *α*, *β*, and *γ*) involved in the free-ion Hamiltonian *H*_0_ (2), for the Er^3+^ ion, are taken from [67,68]. The second-order contributions from the excited CF states, |*i*>, to the *χ_αβ_* tensor of the anisotropic magnetic susceptibility (the second term in Equation (5)) were taken both from the ground *J*-multiplet, ^4^I_15/2_, and the several excited multiplets of the Er^3+^ ion, (^4^I_13/2_, ^4^I_11/2_, ^4^I_9/2_). Special care is taken with the rank two (*k* = 2) *B_kq_* parameters, which are the most responsible for the magnetic anisotropy. These CF parameters are sensitive to the long-range interactions, whose range is beyond the coordination polyhedron of the Er^3+^ ion; therefore, they are not correctly described by the superposition CF model. For this reason, we apply refined CF calculations, in which the rank two *B*_2*q*_ parameters are varied instead of the *b*_2_ “intrinsic” CF parameter for the O, N, and Cl atoms. Numerical calculations are performed with routines described in [69,70,71].

The best fit to the experimental *χT* curves of **2**–**5** (Figure 7) is reached at the *b*_4_ and *b*_6_ intrinsic parameters, listed in Table S10; the calculated rank two *B*_2*q*_ parameters are shown in Table S11. Note that a scaling factor for the magnetic susceptibility was applied for Complexes **3** and **4** (11% and 12%, respectively) in order to cover some uncertainty in the lanthanide concentration in the powder samples. The simulated *χT* curves for **2**–**5** match well with the experimental data in the whole temperature range (Figure 7). The results of the CF calculations indicate that the heteroligand pentagonal-bipyramidal coordination of the Er^3+^ ion in **2**–**5** produces a low CF splitting energy of the lowest ^4^*I*_15_*_/_*_2_ multiplet, within 350 cm^−1^ (Table 1). The overall strength of the CF potential is measured by the CF strength criterion, *S*, which is about 600 cm^−1^ or less (see Table S11) [72]. In fact, the low CF splitting energy in **2**–**5** indicates that these PBP erbium complexes are unlikely to be high-performance SMMs because large CF splitting energy is known to be the most important necessary condition to having a high spin-reversal barrier, *U*_eff_.

To obtain more insight into the electronic structure and magnetic properties of the Er^3+^ ions centered in the various PBP coordination polyhedra, we performed ab initio CASSCF/RASSI-SO/SINGLE_ANISO calculations for Complexes **2**–**5**; the computational details are reported below in Section 3.4. The CASSCF/RASSI-SO/SINGLE_ANISO-calculated CF splitting energies (cm^−1^) are reasonably comparable to the CF energy spectrum obtained from the CF analysis for all complexes, except for Complex **5** (Table 1). On the basis of these data, we simulated the *χT (T)* and *M(B)* curves (Figure S12), which are also consistent with the experimental data, especially for the neutral complexes, **2**–**4**. The less favorable results of the quantum chemical calculations for Complex **5** are seemingly due to the negative electric charge of Complex **5**, which differs from the neutral complexes, **2–4**. This fact makes difficult the correct taking into account of the effects of the crystal packing for the charged moieties within the framework of the quantum chemical consideration of an isolated complex. Indeed, for the isostructural Dy analog of Complex **5** [55], there is also a not-so-good agreement between the energy of the first excited KD and the experimental estimation of the spin-reversal barrier, *U_eff_*.

On the other hand, our CF analysis indicates the overall uniaxial nature of the CF potential in **2**–**5**, as can be seen from the fact that the axial CF parameter, *B*_40_, strongly dominates among the other *B_kq_* parameters (Table S11). This CF splitting pattern tends to stabilize the *M_J_* = ±11/2 ground Kramers doublet of the Er^3+^ ion. As a result, the calculated *g*-tensor of the ground state in **2**–**5** has a uniaxial Ising-type character (*g_z_* >> *g_x_*, *g_y_*, Table 1), which generally favors the SMM behavior (see the next section).

#### 2.3.3. Dynamic (AC) Magnetic Properties

**Figure 8:**
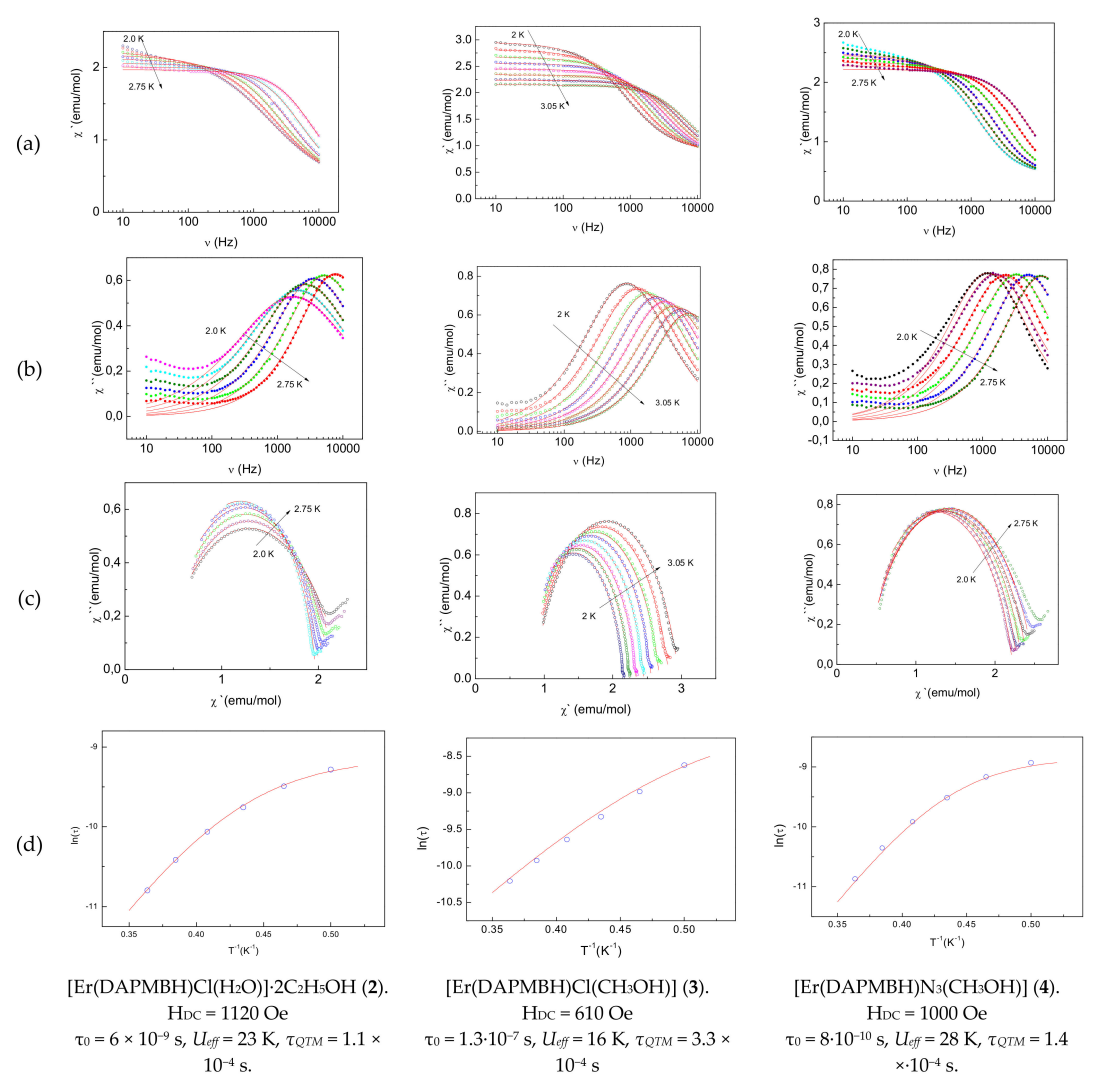
The frequency dependencies of the *χ*′ and *χ*″ under a magnetic field, H_DC_ ((**a**) and (**b**), respectively); (**c**) the Cole–Cole diagram at different temperatures; and (**d**) magnetization relaxation time, *ln(τ)* vs. *T*^−1^, plot for Complexes **2** (left), **3** (center), and **4** (right). The red line represents the fit to Equation 11, accounting for the contribution from QTM and the Orbach relaxation processes with corresponding parameters.

To compare the dynamic magnetic properties of the Er-based complexes, we performed the alternating current magnetic susceptibility (AC) measurements. The in-phase χ′(ν) and the non-zero out-of-phase χ″(ν) magnetic susceptibility of the complexes were measured in the frequency range of 10 Hz–10 kHz, in temperatures of 2 K–4 K in the zero and non-zero magnetic fields. In the studied frequency and temperature ranges, the complexes, [(Et_3_H)N]^+^[Er(H_2_DAPS)Cl_2_]^−^ (**5**), and [(Et_3_H)N]^+^[Y_0_._95_Er_0_._05_(H_2_DAPS)Cl_2_]^−^ (**6**), do not display the frequency dependence of the χ′(ν) and χ″(ν) signals in the zero/non-zero DC applied field, opposite to the similar isostructural Dy complexes, which are field-induced SMMs [53]. A similar behavior was observed in the case of erbium and dysprosium anionic complexes, [Er(Dy)(H_2_DAPS)Cl_2_]^−^, with the counterion, [(CH_3_)_4_N]^+^ [54]. Such a difference in the magnetic properties of these isostructural dysprosium and erbium complexes can be easily understood in terms of the difference in the form of the 4*f* electron density for the Dy^3+^ and Er^3+^ ions: oblate and prolate (equatorially or axially elongated *f*-electron charge clouds), respectively [35]. In these complexes, the presence of two negatively charged ligands (Cl^−^) in the axial positions leads to strong electronic repulsion between the electron density of the Er^3+^ ion and these ligands, which results in the energy destabilization of the prolate Er^3+^ ion, followed by the loss of SMM properties [35,36,37]. More quantitatively, specific reasons for the SMM-silent behavior in **5** and **6** are the presence of significant transverse components in the ground state *g*-tensor (*g_x_* = 2.07, *g_y_* = 4.88, *g_z_* = 12.37), as well as the low-energy positions of the first excited CF states of the Er^3+^ ion (9 and 22 cm^−1^ for the first and second excited CF states, respectively, Table 1), which together result in fast magnetic relaxation. The dilution of erbium with diamagnetic yttrium (Complex **6**) does not lead to the appearance of a frequency dependence of χ″ in the zero/non-zero permanent magnetic field, indicating that the loss of the SMM properties is not associated with dipole–dipole interactions [73,74], but originates exclusively from the crystal field effect. Replacement of one negatively charged ligand in the apical positions with one neutral ligand (H_2_O or CH_3_OH) leads to Complexes **2**–**4**, which exhibit SMM behavior in a permanent magnetic field. The magnitude of the magnetic field was set at a value corresponding to the position of the maximum in the magnetic field dependence of the out-of-phase part of magnetic susceptibility, χ″(*B*), at 2 K and a fixed frequency AC excitation (Figure S13). As can be seen from Figure 8, the shape and appearance of the χ′(ν) and χ″(ν) frequency dependences for Complexes **2**–**4** differ, but the position of the maximum in the χ″(ν) shows a similar dynamic: the peak quickly shifts towards higher frequencies with increasing temperature, and already exceeds the 10 kHz threshold at 3 K, thereby signifying the considerable impact from the phonon-assisted magnetic relaxation processes. To get more insight into the peculiarities of the relaxation of magnetization in these complexes, the observed experimental frequency dependencies of the AC magnetic susceptibilities were analyzed within the framework of the generalized Debye model (Equations (8)–(10)):

(8) $$\chi_{total}\left(\omega \right)=\chi_{s}+\frac{\left(\chi_{T}-\chi_{S}\right)}{1+{\left(i\omega \tau \right)}^{1-\alpha }}$$

(9) $$\chi \prime \left(\omega \right)\;=\;\chi_{s}+\left(\chi_{T}-\chi_{S}\right)\frac{\left(1+{\left(\omega \tau \right)}^{1-\alpha }\sin\left(\frac{\pi \alpha }{2}\right)\right)}{1+2{\left(\omega \tau \right)}^{1-\alpha }\sin\left(\frac{\pi \alpha }{2}\right)+{\left(\omega \tau \right)}^{2\left(1-\alpha \right)}}$$

(10) $$\chi \prime \prime \left(\omega \right)\;=\;\left(\chi_{T}-\chi_{S}\right)\frac{{\left(\omega \tau \right)}^{1-\alpha }\cos\left(\frac{\pi \alpha }{2}\right)}{1+2{\left(\omega \tau \right)}^{1-\alpha }\sin\left(\frac{\pi \alpha }{2}\right)+{\left(\omega \tau \right)}^{2\left(1-\alpha \right)}}$$

where parameter α is a measure of the distribution of the relaxation time that characterizes the deviation from the pure Debye process; $\chi_{s}$, $\chi_{T}$, are the adiabatic and isothermal susceptibilities, respectively; ω is the angular frequency; and *τ* is the relaxation time.

In a low-frequency range, the out-of-phase AC magnetic susceptibility, χ″(ν), for Complexes **2** and **4** decreases with a frequency increase from 10 Hz to 150 Hz. This low-frequency signal may signify the presence of a second relaxation of the magnetization process with slower dynamics. The fitting of the experimental data was performed in the frequency range of 200 Hz–10 000 Hz; the resulting curves and parameters obtained for the three complexes are presented in Figure 8 and Tables S12–S14. The semicircle-like shape of the Cole–Cole plots corresponds to the values of the parameter, α, ranging from 0.35–0.13, 02–0.11, and 0.18–0.16 for **2**, **3,** and **4**, respectively (see Tables S12–S14). The non-zero values of α indicate the multiple relaxation paths contributing to the reversal of the magnetization process. The fit of the temperature dependences of the relaxation times for the three complexes were carried out using Equation (11), accounting for the various relaxation processes:

(11) $$\tau^{-1}=\tau_{QTM}^{-1}+AT+CT^{n}+\tau_{0}^{-1}\exp\left(-\frac{U_{eff}}{kT}\right)$$

where *T* is the absolute temperature; *U*_eff_ is the effective energy barrier for the reversal of magnetization; *k* is the Boltzmann constant. The $\tau_{QTM}^{-1}$ term in (11) represents the temperature-independent contribution from the quantum tunneling of the magnetization (QTM) effects, the second and third terms are direct relaxation and the Raman process, respectively; the exponential term describes the thermally activated mechanism of magnetic relaxation (the Orbach process).

The analysis of the temperature dependences of the relaxation time points to the dominance of QTM and Orbach relaxation mechanisms in all three complexes, displaying the barriers, *U*_eff_ = 23 K, 16 K, and 28 K for **2**, **3**, and **4**, respectively (Figure 8d). The comparable values of *U*_eff_ in **2**–**4** are consistent with the fact that the Er^3+^ ions in all three complexes are in a similar coordination geometry, produced by the same equatorial ligand (DAPMBH) and mixed axial ligands involving one charged group (Cl^−^, N_3_^−^) and one neutral molecule (H_2_O, CH_3_OH).

The difference in the SMM behavior between Complexes **2**–**4** and **5** can straightforwardly be explaned in light of our CF calculations, which indicate considerably larger energy of the first excited CF state in **2**–**4** (22–32 cm^−1^), as compared to that in **5** (9 cm^−1^), as well as less pronounced transverse components (*g_x_*, *g_y_*) in the ground-state *g*-tensor (Table 1). Recently, a neutral PBP Er complex, with an acyclic pentadentate (*N_3_O_2_*) ligand, H_2_DAPBH (see Scheme 1) in the equatorial plane and axial ligands, Cl^-^, and neutral tricyclohexylphosphine oxide was synthesized [55]. Unlike our Er complexes (**2**–**4**), and similarly to Complex **5,** this complex is not a field-induced single-ion magnet, as it does not show the frequency dependence of the χ″(ν) signal in the zero/non-zero DC applied field. As in our Complexes **2** and **3**, this complex has a mixed composition of axial ligands involving one Cl− ligand; however, the replacement of water or alcohol ligands with phosphine oxide results in a loss of the SMM properties. This fact is rather surprising, especially considering the stronger axial field due to the phosphine oxide ligand, which is a stronger donor ligand compared to H_2_O and alcohol (complexes **2**–**4**); a stronger axial field usually improves the SMM performance, as opposed to this case.

## 3. Materials and Methods

The 2,6-diacetylpyridine, Et_3_N, ErCl_3_ (anhydrous), NaN_3_, ethanol (anhydrous), 96% ethanol, 4-methoxybenzoic acid, thionyl chloride, hydrazine hydrate solution (50–60% N_2_H_4_), and salicylhydrazide were purchased from commercial sources and used without further purification. Methanol was dried upon refluxing with magnesium methoxide, followed by distillation.

The infrared spectra were measured on solid samples using a Perkin Elmer Spectrum 100 Fourier Transform infrared spectrometer (Waltham, MA, USA) in the range of 4000–500 cm^−1^.

Elemental analyses were carried out by the Analytical Department Service at the Institute of Problems of Chemical Physics, RAS, using a vario MICRO cube (Elementar Analysensysteme GmbH) equipment.

Both the AC and DC magnetic properties were measured using the Physical Properties Measurements System PPMS-9 (Quantum Design), in the temperature range of T = 2–300 K, under a magnetic field up to B = 7 T. The field dependences of the magnetization were measured at several temperatures between 2 and 7 K, with a DC magnetic field up to 7 T. The samples, in polycrystalline form, were loaded into an insulating capsule. The experimental data were corrected for the sample holder. The diamagnetic contribution from the ligand was calculated using Pascal’s constants.

### 3.1. Synthesis and Characterization

Ligands [H_2_DAPMBH] and [H_4_DAPS] were synthesized according to the published procedures [40,43,56].

[Er(DAPMBH)(C_2_H_5_OH)Cl] (**1**). To a suspension of H_2_DAPMBH (0.74 mmol, 338 mg) in absolute ethanol (37 mL), a solution of ErCl_3_ in absolute ethanol (0.74 mmol, 202 mg in 33 mL C_2_H_5_OH) was added using a dropping funnel at r.t. The white suspension turned to yellow immediately, and the white precipitate started to dissolve. The reaction mixture was stirring at 60 °C for one hour. During this time, the white precipitate of the ligand was completely dissolved and a large amount of a light-yellow solid appeared. After that, the reaction mixture was cooled to room temperature and triethylamine (0.22 mL, 1.55 mmol) was added under stirring. As a result, within about 5 min, the light-yellow precipitate completely dissolved and, instead, an abundant fine-crystalline bright-yellow precipitate began to precipitate. The reaction mixture was stirring at 60 °C for another 15 min and was then cooled to room temperature. After cooling, the mother liquor was decanted from the precipitate, which was washed with diethyl ether (2 × 7 mL) and dried in vacuo, affording 0.39 g of Product **1**. Yield 75%. Anal. Calcd. for C_27_H_29_ClErN_5_O_5_ (706.27): C, 45.92; H, 4.11; N, 9.92%. Found: C, 46.13; H, 4.34; N, 9.72%. FT-IR (solid, in cm^−1^): 1604 (m), 1497 (m), 1363 (vs), 1250 (s), 1168 (vs), 1028 (s), 847 (m), 764 (s), and 621 (m) (Figure S14).

[Er(DAPMBH)(H_2_O)Cl]∙2C_2_H_5_OH (**2**). To a suspension of H_2_DAPMBH (0.61 mmol, 280 mg) in 96% ethanol (30 mL), a solution of ErCl_3_ in ethanol (0.61 mmol, 167 mg in 27 mL C_2_H_5_OH) was added using a dropping funnel at r.t. The white suspension turned to yellow immediately, and the white precipitate started to dissolve. The reaction mixture was stirring at 60 °C for one hour. During this time, the white precipitate of the ligand was completely dissolving, and a lot of a yellow precipitate appeared. After that, the reaction mixture was cooling to room temperature and triethylamine (0.18 mL, 1.3 mmol) was adding under stirring. After about 15 min of stirring the reaction mixture at 60 °C, the precipitate dissolved, and the solution became more a saturated bright yellow. The reaction mixture was stirring at 60 °C for another 15 min and then cooled to room temperature. The solution was filtered and left to evaporate the solvent at room temperature. Crystal formation was observed within 2 weeks, with significant evaporation of the solution. The mother liquor was decanted from crystals, which were washed with diethyl ether (2 × 7 mL) and dried in vacuo, affording 0.18 g of Product **2**. Yield 39%. Anal. Calcd. for C_29_H_37_ClErN_5_O_7_ (770.35): C, 45.22; H, 4.81; N, 9.10%. Found: C, 44.83; H, 4.54; N, 9.42%. FT-IR (solid, in cm^−1^): 1606 (m), 1552 (m), 1495 (m), 1370 (vs), 1256 (s), 1165 (vs), 1028 (s), 994 (s), 855 (m), 764 (s), and 620 (m).

[Er(DAPMBH)(CH_3_OH)Cl] (**3**). **1** (0.17 mmol, 120 mg) was dissolved in 5 mL of absolute CH_3_OH. The solution was filtered and left to slowly evaporate the solvent at room temperature. After a few days, the mother liquor was decanting from the precipitated crystals; the crystals were washed with ether (2 × 3 mL) and dried in vacuo. Yield 85%. Anal. Calcd. for C_26_H_27_ClErN_5_O_5_ (692.24): C, 45.12; H, 3.90; N, 10.12%. Found: C, 45.43; H, 4.14; N, 9.92%. FT-IR (solid, in cm^−1^): 1605 (m), 1552(m), 1499 (m), 1365 (vs), 1255 (s), 1168 (vs), 1028 (s), 850 (m), 764 (s), and 621 (m).

[Er(DAPMBH)(CH_3_OH)(N_3_)] (**4**). **1** (0.14 mmol, 100 mg) was dissolved in 10 mL of absolute methanol and the solid NaN_3_ (10 mmol, 65 mg) was added to the reaction mixture, and the solution was stirring at 60 °C for about 30 min. After the cooling and filtration of a small precipitate, the filtrate was left standing undisturbed at r. t. to slowly evaporating the solvent. Bright-yellow crystals of **4**, suitable for X-ray diffraction, precipitated in the course of several days. The mother liquor was decanting from crystals, which were washed first with cold methanol (1 × 3 mL), and then with diethyl ether (2 × 3 mL), and dried in vacuo, affording 59 mg of Product **4**. Yield 60%. Anal. Calcd. for C_26_H_27_ErN_8_O_5_ (698.81): C, 44.70; H, 3.87; N, 16.05%. Found: C, 44.33; H, 4.14; N, 15.72%. FT-IR (solid, in cm^−1^): 2091 (vs, ν_azide_), 1607 (m), 1553 (m), 1401 (m), 1365 (vs), 1252 (s), 1167 (vs), 1025 (s), 1008 (m), 764 (s), and 621 (m) (Figure S15). 

[(Et_3_H)N]^+^[Er(H_2_DAPS)Cl_2_]^−^ (**5**). To a suspension of H_2_DAPS (0.46 mmol, 200 mg) in absolute ethanol (20 mL), a solution of ErCl_3_ in ethanol (0.46 mmol, 126 mg in 10 mL C_2_H_5_OH) was added at r.t. The white suspension turned to yellow immediately, and the white precipitate started to dissolve. The reaction mixture was refluxed for 1h under stirring conditions. After that, it was cooled to room temperature and triethylamine (0.14 mL, 1 mmol) was adding under stirring. The reaction mixture became more a saturated bright-yellow after about 5–10 min of stirring. The solution was filtered and left to evaporate the solvent at room temperature. Crystal formation was observed within 3–5 days, with significant evaporation of the solution. The mother liquor was decanted from crystals, which were washed with diethyl ether and dried in vacuo, affording 180 mg of Product **5**. Yield 51%. Anal. Calcd. for C_29_H_35_N_6_O_4_Cl_2_Er (769.8): C, 45.24; H, 4.55; N, 10.92%. Found: C, 45.66; H, 4.81; N, 10.93%. FT-IR ν_max_/cm^−1^: 3166 m, 3084 m, 1646 s, 1640 s, 1607 m, 1549 s, 1493 vs, 1453 m, 1361 m, 1302 m, 1230 vs, 1163 vs, 1083 m, 1039 m, 990 m, 919 m, 817 vs, and 776 m.

[(Et_3_H)N]^+^[Y_0_._95_Er_0_._05_(H_2_DAPS)Cl_2_]^−^ (**6**). To a suspension of H_2_DAPS (0.46 mmol, 200 mg) in absolute ethanol (20 mL), a solution of ErCl_3_ (0.03 mmol, 8 mg) and YCl_3_ (0.43 mmol, 181 mg) in 10 mL of ethanol was added at r.t. The white suspension turned to yellow immediately, and the white precipitate started to dissolve. The reaction mixture was refluxed for 1h under stirring conditions. After that, it was cooled to room temperature and triethylamine (0.14 mL, 1 mmol) was adding under stirring. The reaction mixture became a more saturated bright yellow after about 5–10 min of stirring. The solution was filtered and left to evaporate the solvent at room temperature. Crystal formation was observed within 3–5 days, with significant evaporation of the solution. The mother liquor was decanted from crystals, which were washed with diethyl ether and dried in vacuo, affording 160 mg of Product **6**. Yield 45%. Anal. Calcd. for C_29_H_35_N_6_O_4_Cl_2_Er_0_._95_Y_0_._05_ (765.88): C, 45.48; H, 4.61; N, 10.97%. Found: C, 45.74; H, 4.89; N, 10.95%.

### 3.2. Crystal Structure Determination

X-ray single crystal diffraction data for Complexes **2**, **3**, **5,** and **6** were collected at 100–150 K on Oxford Diffraction CCD diffractometers [λ(Mo*K*_α_) = 0.71073 Å, graphite monochromator, ω-scans]. Single crystals were taken from the mother liquid using a nylon loop with paratone oil and immediately transferred into the cold nitrogen stream of the diffractometer. Data reduction with the empirical absorption correction of the experimental intensities (Scale3AbsPack program) was made with the CrysAlisPro software [75].

X-ray diffraction data for Complex **4** were collected on the “Belok” beamline of the Kurchatov Synchrotron Radiation Source (National Research Center “Kurchatov Institute”, Moscow, Russian Federation), in the *ϕ*-scan mode using a Rayonix SX165 CCD detector at 100 K, λ = 0.78790 Å [76]. The data were indexed, integrated, scaled, and corrected for absorption using the XDS program package [77].

The structures were solved by a direct method and refined by a full-matrix least squares method against the *F*^2^ data with anisotropic displacement parameters for all the nonhydrogen atoms using SHELX programs [78]. The H-atoms were refined in a riding model with isotropic displacement parameters, depending on the *U*_eq_ of the connected atom. The O–H bond distances were refined in the H_2_O and MeOH axial ligands of the Er complexes. Position disorder was found in Structure **2** (EtOH solvent molecules). The selected crystallographic parameters and the refinement statistics for **2**–**6** are given in Table S15. The crystallographic data have been deposited with the Cambridge Crystallographic Data Center (deposit@cc.cam.ac.uk, http://www.ccdc.cam.ac.uk/data_request/cif (accessed on 13 July 2021)), and the CCDC reference codes are listed in Table S15.

### 3.3. Simulation of Static Magnetic Properties and CF Calculations

Crystal field (*CF*) analysis for Complexes **2**–**5** was carried out with the conventional CF theory for f-electrons, based on the Wybourne parameterization scheme [60,61,62], in combination with the superposition CF model [64,65,66] adapted for low-symmetry metal sites. Simulation of the magnetic susceptibility was performed in terms of the Gerloch–McMeeking equation [63], using computational routines described elsewhere [69,70,71].

### 3.4. Computational Details

The ab initio calculations for Complexes **2**–**5** were performed using the *OpenMolcas* program [79,80]. The [.ANO-RCC...8s7p5d3f2g1h.] basis set for the Er atom, the [.ANO-RCC...3s2p1d.] for the Cl, N, and O atoms, [.ANO-RCC...3s2p.] for the C atoms, and [.ANO-RCC...2s.] for the H atoms have been employed. All calculations were based on the experimental geometries from the X-ray single crystal diffraction. The ground state f-electron configuration for Er(III) is 4f^11^, having the *^4^I_15/2_* multiplet as a ground state. Initially, we have generated the guess orbitals from where the seven Er(III)-based starting orbitals were selected to perform the CASSCF calculations, where 11 electrons are in the 7 active orbitals with an active space of CAS (11,7). Using this active space, 35 quartets and 112 doublets, using the configuration interaction (CI) procedure, have been computed. After that, all these 35 quartets and all 112 doublets using the RASSI-SO module were mixed to compute the spin–orbit states. After computing these spin–orbit states, using the *SINGLE_ANISO* code [81], the corresponding g-tensors and the CF parameters for the eight low-lying Kramers doublets (KD) were extracted. The Cholesky decomposition for the two electron integrals was employed throughout in the calculations to reduce the disk space. The second-order Douglas–Kroll–Hess [82,83,84,85] scalar relativistic Hamiltonian was used to treat the scalar relativistic effects. 

## 4. Discussion and Conclusions

A series of six erbium complexes, with acyclic pentadentate (*N_3_O_2_*) ligands (H_2_DAPMBH and H_4_DAPS) in the equatorial plane, and charged (Cl^−^, N_3_^−^) and neutral ligands (C_2_H_5_OH, H_2_O, CH_3_OH) in the apical positions were synthesized: [Er(DAPMBH)(C_2_H_5_OH)Cl] (**1**); [Er(DAPMBH)(H_2_O)Cl] 2C_2_H_5_OH (**2**); [Er(DAPMBH)(CH_3_OH)Cl] (**3**); [Er(DAPMBH)(CH_3_OH)(N_3_)] (**4**); [(Et_3_H)N]^+^[Er(H_2_DAPS)Cl_2_]^−^ (**5**); and [(Et_3_H)N]^+^ [Y_0_._95_Er_0_._05_(H_2_DAPS)Cl_2_]^−^ (**6**). Depending on the synthesis conditions, neutral (**1**–**4**) and anionic (**5**, **6**) complexes were obtained. It is interesting to note that the attempts to synthesize the neutral Dy complexes, analogous to Complexes **1**–**4**, were unsuccessful; in these cases, anionic complexes similar to Complex **5** were formed. The X-ray diffraction analysis of Complexes **2**–**6** showed that, in all of them, the coordination polyhedron was close to the pentagonal bipyramid, as established by the analysis of their structures with the Shape Program. Compounds **2**–**6** contain well-isolated metal complexes, which are connected to each other and to counterions by hydrogen bonding and π-stacking. Hydrogen-bonded centrosymmetric dimers are found in Structures **2** and **4**. The influence of the charge state of the axial ligands on the magnetic properties of Complexes **2**–**6** was explored: it was found that Complexes **2**–**4,** with one charged and one neutral axial ligand, are field-induced SMMs with the energy barriers, *U*_eff_ ≈ 16–28 K, while Complexes **5** and **6,** with two negative axial ligands (Cl^−^), are SMM-silent. The effective magnetization barriers, *U*_eff_, for Complexes **2**–**4** are fairly consistent with the results of crystal field and ab initio calculations of the electronic structure of the Er^3+^ ions in these complexes. The compounds are the first field-induced single-ion magnets among the known pentagonal-bipyramidal Er complexes with acyclic *N_3_O_2_* Schiff-base ligands. The SMM behavior of Complexes **2**–**4** can be primarily attributed to the strong axiality of the crystal field produced by the PBP ligand environment, as can be seen from the fact that the axial CF term, *B*_40_ (>1000 cm^−1^), strongly dominates over other CF terms, *B_kq_* (<400 cm^−1^, Table S11). The CF analysis for the series of Compounds **2**–**5** reveals rather small variations of the largest nonaxial terms, *B_kq_* (*q* ≠ 0) (*B*_44_, *B*_64_ and *B*_66_), arising from the equatorial pentadentate ligand (Table S11), in accordance with insignificant changes in the geometry of the *N_3_O_2_* chelate ring. On the other hand, the leading axial term, *B*_40_, is sensitive to the charge state and the nature of the axial ligands (Table S11). The largest values of *B*_40_ (about 1600 cm^−1^) occur in Compounds **2** and **3**, which result in larger total CF splitting energy and the overall CF strength, measured by the *S* criterion (Table S11); this correlates with the results of the ab initio calculations (Table 1). The CF calculations indicate the Ising-type character of the ground-state *g*-tensor, which favors reduced QTM and slow magnetic relaxation; surprisingly enough, ab initio calculations result in large transverse *g*-tensor components, *g_x_* and *g_y_*, which are incompatible with the SMM behavior (Table 1). The absence of SMM properties in Complex **5,** with two negatively charged apical ligands (Cl^−^), is most likely because of the larger nonaxiality of the ground-state *g*-tensor (*g_x_* = 2.07, *g_x_* = 4.88 *g_x_* = 12.37, Table 1), as compared to that in Compounds **2**–**4**. Interestingly, ab initio calculations again lead to the opposite results for Complex **5,** resulting in the largest *g*-tensor axiality in the series of Compounds **2**–**5** (Table 1). One more reason for the SMM-silent behavior of **5** is the presence of a low-lying Kramers doublet (at 9 cm^−1^) with very strong nonaxiality (*g_x_* = 2.70, *g_y_* = 6.34, *g_z_* = 7.75, Table 1), causing fast thermally activated QTM. This is consistent with the fact that the dilution of Er with diamagnetic Y (Complex **6**) does not lead to the appearance of χ″ frequency dependence, even in a DC field. 

## Figures and Tables

**Table 1:** Calculated CF splitting energies (cm^−1^) of the lowest ^4^I_15/2_ multiplet of the Er^3+^ ion and *g*-tensors of the ground, and first excited CF states in erbium complexes, **2**–**5** (Appendix A), based on the fitting to the DC magnetic data (left), and the ab initio calculations (right).

2		3		4		5	
0	0.00	0	0.00	0	0.00	0	0.00
29.2	33.17	32.1	35.87	21.9	26.29	9	26.24
49.7	54.6	62.4	60.3	50.5	44.58	22	60.15
99	84.29	95.9	96.98	69.9	92.06	74.1	159.71
197.8	174.94	182.9	190.25	146	233.03	130.3	199.31
272.8	307.39	293.2	286.1	185.5	288.5	203.7	281.43
301.2	452.97	310	441.15	211.9	409.12	207.8	399.21
321.6	488.93	336.2	472.05	278.7	429.09	245.5	423.48
*g*-tensor components of the ground CF state							
*g_x_* = 0.53	0.031	*g_x_* = 1.74	0.353	*g_x_* = 0.09	0.458	*g_x_* = 2.07	0.439
*g*_y_ = 2.10	5.166	*g_y_* = 1.98	3.347	*g_y_* = 2.02	4.697	*g_y_* = 4.88	0.547
*g_z_* = 13.44	10.9	*g_z_* = 13.95	12.954	*g_z_* = 14.20	11.512	*g_z_* = 12.37	15.04
*g*-tensor components of the first excited CF state							
*g_x_* = 1.96	0.348	*g_x_* = 2.15	0.914	*g_x_* = 2.75	0.925	*g_x_* = 2.70	7.308
*g_y_* = 5.09	2.642	*g_y_* = 5.61	3.008	*g_y_* = 5.24	1.799	*g_y_* = 6.34	6.727
*g_z_* = 9.68	6.157	*g_z_* = 9.18	7.296	*g_z_* = 8.92	5.048	*g_z_* = 7.75	4.135

## Data Availability

The data presented in this study are available in the present article and the Supplementary Information Section.

## Supplementary Materials

Figure S1: Asymmetric unit with atom numbering scheme in **2** (30% thermal ellipsoids, H atoms are omitted for clarity). Occupancy of disordered EtOH solvent molecules: O1Sa–0.8, O1Sb–0.2, O2Sa–0.6, O2Sb–0.2, O3Sa–0.2.; Figure S2: (**a**) The *ab* layer of Er complexes in **2**. O-H…N, O/C-H…O, O/C-H…Cl contacts are shown by blue, red, and green dashed lines, respectively. The shortest Er…Er separations (brown dotted lines) are 7.0386(4) Å (1, dimer), 8.3532(4) Å (2) and 8.5853(4) Å (3). (**b**) Centrosymmetric H-bonded dimer in **2**. C…C contacts < 3.6 Å are shown by black dotted lines; Figure S3: Asymmetric unit with atom-numbering scheme in **3** (50% thermal ellipsoids, H atoms are omitted for clarity); Figure S4: (**a**) Infinite chain of hydrogen-bonded Er complexes in **3**. (**b**)View of the AC layer in Structure **3**. Hydrogen bonds (red dashed lines for C-H…O and O-H…Cl, green dashed lines for C-H…Cl), Er…Er distances (brown dashed lines, 1 = 7.0338(2) Å, 2 = 7.6231(5) Å), C…C contacts < 3.6 Å (black dotted lines) are shown; Figure S5: Asymmetric unit with atom-numbering scheme in [Er(DAPMBH)(CH_3_OH)(N_3_)] (**4**) (35% thermal ellipsoids, H atoms are omitted for clarity); Figure S6: Dimeric hydrogen-bonded units in crystal structure of [Er(DAPMBH)(CH_3_OH)N_3_] (**4**). The hydrogen bonds, O-H…N, are shown with blue dotted lines, *π-π* stacking interaction between aromatic systems of the ligands are shown with grey dashed lines. Color code: erbium–green, oxygen–red, nitrogen–blue, carbon–grey. All distances are given in Å; Figure S7: Short intermolecular contacts in crystal structure packing of [Er(DAPMBH)(CH_3_OH)N_3_]. In addition to *π-π* stacking interaction, short contacts between carbon atoms are shown (C…C < 3.6 Å, all distances are given in Å). Azide anions and methanol molecules are omitted for clarity; Figure S8: Fragment of 1D polymeric chain of Complex **4**, mutual arrangement of two doubly hydrogen-bonded units are shown. Most of the hydrogen atoms are omitted for clarity; Figure S9: Asymmetric unit with atom-numbering scheme in **5** (30% thermal ellipsoids, H atoms are omitted for clarity); Figure S10: Unit cell contents in the crystal packing of **5** and **6** along crystallographic *a* (left) and *c* (right) axes. The inter Er–Er and Y–Y distances of the neighbor molecules are shown by green dashed lines (values are in Å). The Et_3_NH counter cations and H atoms are omitted for clarity; Figure S11: Fragment of the crystal packing of **5**. Dotted cyan lines show the intermolecular contacts; Figure S12: Experimental (open circles) and theoretical (solid lines) temperature dependences of magnetic susceptibility (in the form of *χT* vs. *T*) and field dependences of magnetization for complexes **2**–**5**. Theoretical data are obtained from ab initio calculations at the CASSCF/RASSI-SO/SINGLE_ANISO level of theory using the [Open]MOLCAS program; Figure S13: The field dependence of the χ′′ at T = 2 K and the fixed frequency AC excitation for complexes **2** (**a**, ν = 100 Hz), **3** (**b**, ν = 500 Hz) and **4** (**c**, ν = 100 Hz); Figure S14: IR-spectrum of the complex [Er(DAPMBH)(C_2_H_5_OH)Cl] (**1**); Figure S15: IR-spectrum of the complex [Er(DAPMBH)(CH_3_OH)N_3_] (**4**); Table S1: SHAPE * analysis; Table S2: Selected bond lengths (Å) and angles (°) in **2**; Table S3: Hydrogen bond geometry in **2**; Table S4: Selected bond lengths (Å) and angles (°) in **3**; Table S5: Hydrogen bond geometry in **3**; Table S6: Selected bond lengths (Å) and angles (°) in **4**; Table S7: Hydrogen bond geometry in **4**; Table S8: Selected bond lengths (Å) and angles (°) in **5** and **6**; Table S9: Comparison of the bond length values (Å) in Er (or Y in 6) polyhedron in Structures **2**–**6**; Table S10: Calculated intrinsic CF parameters, b_4_(L) and b_6_(L), (where L = O, N, Cl) (in cm^−1^) of the superposition CF model providing the optimum fit to the magnetic susceptibility of Complexes **2**–**5** (Figures S7–S10); Table S11: Calculated CF parameters, *B_kq_* (cm^−1^), corresponding to the optimum fit to the magnetic susceptibility of Complexes **2**–**5** (Figures S7–S10). Real (Re) and imaginary (Im) parts of the complex *B_kq_* parameters are indicated; Table S12: Results of fit of χ″(ν) and χ′(ν) plots by generalized Debye model at 1120 Oe for **2**; Table S13: Results of fit of χ″(ν) and χ′(ν) plots by generalized Debye model at 610 Oe for **3**; Table S14: Results of fit of χ″(ν) and χ′(ν) plots by generalized Debye model at 1000 Oe for **4**; Table S15: Crystal data and structural refinement parameters for Complexes **2**–**6**. CCDC 2107393, 2107394, 2100722, 2099114, and 2100999 contain the supplementary crystallographic data for this paper. These data can be obtained free of charge via www.ccdc.cam.ac.uk/data_request/cif (accessed on 13 July 2021), or by emailing data_request@ccdc.cam.ac.uk, or by contacting The Cambridge Crystallographic Data Centre, 12 Union Road, Cambridge CB2 1EZ, UK; Fax: +44-1223-336033.

## Sample Availability

Samples of compounds are available from the authors.

## Appendix A

Note here that the data obtained from detailed high-frequency/high-field electron paramagnetic resonance (HF-EPR) studies of **5** are in good agreement with the results from CF analysis for the Er(III) ion based on the simulation of the *DC* magnetic data of **5**. *See* Lena Spillecke, Changhyun Koo, Olga Maximova, Vladimir S. Mironov, Vyacheslav A. Kopotkov, Denis V. Korchagin, Alexander N. Vasiliev, Eduard B. Yagubskii, and Rüdiger Klingeler, “Magnetic behavior of the novel pentagonal-bipyramidal Erbium(III) complex (Et_3_NH)[(H_2_DAPS)ErCl_2_]: high-frequency EPR study and crystal-field analysis”. The paper pending in *Dalton Transactions*.
