# Supplementary material for: A Series of Novel Pentagonal-Bipyramidal Erbium(III) Complexes with Acyclic Chelating N3O2 Schiff-Base Ligands: Synthesis, Structure, and Magnetism
Source: Molecules. 2021 Nov 16;26(22):6908. doi: 10.3390/molecules26226908 (PMC8622354; doi:10.3390/molecules26226908)

checkCIF/PLATON report

Structure factors have been supplied for datablock(s) tb11

THIS REPORT IS FOR GUIDANCE ONLY. IF USED AS PART OF A REVIEW PROCEDURE FOR PUBLICATION, IT SHOULD NOT REPLACE THE EXPERTISE OF AN EXPERIENCED CRYSTALLOGRAPHIC REFEREE.

No syntax errors found. CIF dictionary Interpreting this report

Datablock: tb11

Bond precision: C-C = 0.0063 A Wavelength=0.71073

Cell:	a=8.3532(2)	b=13.8859(2)	c=14.6874(4)
	alpha=88.386(2)	beta=80.033(2)	gamma=83.244(2)
Temperature:	120 K		

	Calculated	Reported
Volume	1666.20(7)	1666.20(7)
Space group	P -1	P -1
Hall group	-P 1	-P 1
Moiety formula	C25 H25 Cl Er N5 O5, 2(C2 H6 O)	C25 H25 Cl Er N5 O5, 2(C2 H6 O)
Sum formula	C29 H37 Cl Er N5 O7	C29 H37 Cl Er N5 O7
Mr	770.35	770.34
Dx,g cm-3	1.536	1.535
Z	2	2
Mu (mm-1)	2.648	2.648
F000	774.0	774.0
F000'	774.07	
h,k,lmax	12,20,21	12,19,21
Nref	10830	9018
Tmin,Tmax	0.535,0.728	0.889,1.000
Tmin'	0.524	

Correction method= # Reported T Limits: Tmin=0.889 Tmax=1.000
AbsCorr = MULTI-SCAN

Data completeness= 0.833 Theta(max)= 31.227

R(reflections)= 0.0447(7803) wR2(reflections)= 0.1120(9018)

S = 1.003 Npar= 484

The following ALERTS were generated. Each ALERT has the format
test-name_ALERT_alert-type_alert-level.
Click on the hyperlinks for more details of the test.

Alert level B

```
PLAT420_ALERT_2_B D-H Bond Without Acceptor  O2SA     --H2SA     .     Please Check
PLAT971_ALERT_2_B Check Calcd Resid. Dens.  0.89A   From Er1             2.98 eA-3
```

Alert level C

```
PLAT250_ALERT_2_C Large U3/U1 Ratio for Average U(i,j) Tensor ....          2.1 Note
PLAT911_ALERT_3_C Missing FCF Refl Between Thmin & STh/L=    0.600          87 Report
PLAT972_ALERT_2_C Check Calcd Resid. Dens.  0.79A   From Er1            -1.81 eA-3
PLAT972_ALERT_2_C Check Calcd Resid. Dens.  0.72A   From Er1            -1.79 eA-3
PLAT972_ALERT_2_C Check Calcd Resid. Dens.  0.58A   From Er1            -1.72 eA-3
PLAT973_ALERT_2_C Check Calcd Positive Resid. Density on       Er1       1.07 eA-3
PLAT975_ALERT_2_C Check Calcd Resid. Dens.  0.46A   From O3SA            1.21 eA-3
```

Alert level G

```
PLAT002_ALERT_2_G Number of Distance or Angle Restraints on AtSite          15 Note
PLAT003_ALERT_2_G Number of Uiso or Uij Restrained non-H Atoms ...          15 Report
PLAT007_ALERT_5_G Number of Unrefined Donor-H Atoms .............            5 Report
PLAT083_ALERT_2_G SHELXL Second Parameter in WGHT  Unusually Large        6.93 Why ?
PLAT154_ALERT_1_G The s.u.'s on the Cell Angles are Equal ..(Note)       0.002 Degree
PLAT172_ALERT_4_G The CIF-Embedded .res File Contains DFIX Records           1 Report
PLAT175_ALERT_4_G The CIF-Embedded .res File Contains SAME Records           3 Report
PLAT178_ALERT_4_G The CIF-Embedded .res File Contains SIMU Records           2 Report
PLAT186_ALERT_4_G The CIF-Embedded .res File Contains ISOR Records           2 Report
PLAT232_ALERT_2_G Hirshfeld Test Diff (M-X)  Er1      --Cl1      .         5.8 s.u.
PLAT300_ALERT_4_G Atom Site Occupancy of O1SA      Constrained at          0.8 Check
PLAT300_ALERT_4_G Atom Site Occupancy of C1SA      Constrained at          0.8 Check
PLAT300_ALERT_4_G Atom Site Occupancy of C2SA      Constrained at          0.8 Check
PLAT300_ALERT_4_G Atom Site Occupancy of H1SA      Constrained at          0.8 Check
PLAT300_ALERT_4_G Atom Site Occupancy of H1S1      Constrained at          0.8 Check
PLAT300_ALERT_4_G Atom Site Occupancy of H1S2      Constrained at          0.8 Check
PLAT300_ALERT_4_G Atom Site Occupancy of H2S1      Constrained at          0.8 Check
PLAT300_ALERT_4_G Atom Site Occupancy of H2S2      Constrained at          0.8 Check
PLAT300_ALERT_4_G Atom Site Occupancy of H2S3      Constrained at          0.8 Check
PLAT300_ALERT_4_G Atom Site Occupancy of O2SA      Constrained at          0.6 Check
PLAT300_ALERT_4_G Atom Site Occupancy of C3SA      Constrained at          0.6 Check
PLAT300_ALERT_4_G Atom Site Occupancy of C4SA      Constrained at          0.6 Check
PLAT300_ALERT_4_G Atom Site Occupancy of H2SA      Constrained at          0.6 Check
PLAT300_ALERT_4_G Atom Site Occupancy of H3S1      Constrained at          0.6 Check
PLAT300_ALERT_4_G Atom Site Occupancy of H3S2      Constrained at          0.6 Check
PLAT300_ALERT_4_G Atom Site Occupancy of H4S1      Constrained at          0.6 Check
PLAT300_ALERT_4_G Atom Site Occupancy of H4S2      Constrained at          0.6 Check
PLAT300_ALERT_4_G Atom Site Occupancy of H4S3      Constrained at          0.6 Check
PLAT300_ALERT_4_G Atom Site Occupancy of O1SB      Constrained at          0.2 Check
PLAT300_ALERT_4_G Atom Site Occupancy of C1SB      Constrained at          0.2 Check
PLAT300_ALERT_4_G Atom Site Occupancy of C2SB      Constrained at          0.2 Check
PLAT300_ALERT_4_G Atom Site Occupancy of H1SB      Constrained at          0.2 Check
PLAT300_ALERT_4_G Atom Site Occupancy of H1S3      Constrained at          0.2 Check
PLAT300_ALERT_4_G Atom Site Occupancy of H1S4      Constrained at          0.2 Check
```

```
PLAT300_ALERT_4_G Atom Site Occupancy of H2S4       Constrained at        0.2 Check
PLAT300_ALERT_4_G Atom Site Occupancy of H2S5       Constrained at        0.2 Check
PLAT300_ALERT_4_G Atom Site Occupancy of H2S6       Constrained at        0.2 Check
PLAT300_ALERT_4_G Atom Site Occupancy of O2SB       Constrained at        0.2 Check
PLAT300_ALERT_4_G Atom Site Occupancy of C3SB       Constrained at        0.2 Check
PLAT300_ALERT_4_G Atom Site Occupancy of C4SB       Constrained at        0.2 Check
PLAT300_ALERT_4_G Atom Site Occupancy of H2SB       Constrained at        0.2 Check
PLAT300_ALERT_4_G Atom Site Occupancy of H3S3       Constrained at        0.2 Check
PLAT300_ALERT_4_G Atom Site Occupancy of H3S4       Constrained at        0.2 Check
PLAT300_ALERT_4_G Atom Site Occupancy of H4S4       Constrained at        0.2 Check
PLAT300_ALERT_4_G Atom Site Occupancy of H4S5       Constrained at        0.2 Check
PLAT300_ALERT_4_G Atom Site Occupancy of H4S6       Constrained at        0.2 Check
PLAT300_ALERT_4_G Atom Site Occupancy of O3SA       Constrained at        0.2 Check
PLAT300_ALERT_4_G Atom Site Occupancy of C5SA       Constrained at        0.2 Check
PLAT300_ALERT_4_G Atom Site Occupancy of C6SA       Constrained at        0.2 Check
PLAT300_ALERT_4_G Atom Site Occupancy of H3SA       Constrained at        0.2 Check
PLAT300_ALERT_4_G Atom Site Occupancy of H5S1       Constrained at        0.2 Check
PLAT300_ALERT_4_G Atom Site Occupancy of H5S2       Constrained at        0.2 Check
PLAT300_ALERT_4_G Atom Site Occupancy of H6S1       Constrained at        0.2 Check
PLAT300_ALERT_4_G Atom Site Occupancy of H6S2       Constrained at        0.2 Check
PLAT300_ALERT_4_G Atom Site Occupancy of H6S3       Constrained at        0.2 Check
PLAT302_ALERT_4_G Anion/Solvent/Minor-Residue Disorder (Resd  2  )       100% Note
PLAT302_ALERT_4_G Anion/Solvent/Minor-Residue Disorder (Resd  3  )       100% Note
PLAT302_ALERT_4_G Anion/Solvent/Minor-Residue Disorder (Resd  4  )       100% Note
PLAT302_ALERT_4_G Anion/Solvent/Minor-Residue Disorder (Resd  5  )       100% Note
PLAT302_ALERT_4_G Anion/Solvent/Minor-Residue Disorder (Resd  6  )       100% Note
PLAT304_ALERT_4_G Non-Integer Number of Atoms in ..... (Resd  2  )       7.20 Check
PLAT304_ALERT_4_G Non-Integer Number of Atoms in ..... (Resd  3  )       5.40 Check
PLAT304_ALERT_4_G Non-Integer Number of Atoms in ..... (Resd  4  )       1.80 Check
PLAT304_ALERT_4_G Non-Integer Number of Atoms in ..... (Resd  5  )       1.80 Check
PLAT304_ALERT_4_G Non-Integer Number of Atoms in ..... (Resd  6  )       1.80 Check
PLAT413_ALERT_2_G Short Inter XH3 .. XHn     H20      ..H4S5     .       2.09 Ang.
                                                        x,y,z  =      1_555 Check
PLAT413_ALERT_2_G Short Inter XH3 .. XHn     H25C     ..H2S5     .       2.12 Ang.
                                                     -1+x,y,z  =      1_455 Check
PLAT432_ALERT_2_G Short Inter X...Y Contact  O3       ..C4SB             2.61 Ang.
                                                    1+x,1+y,z  =      1_665 Check
PLAT432_ALERT_2_G Short Inter X...Y Contact  C1       ..C4SB             2.91 Ang.
                                                    1+x,1+y,z  =      1_665 Check
PLAT720_ALERT_4_G Number of Unusual/Non-Standard Labels ..........         45 Note
PLAT789_ALERT_4_G Atoms with Negative _atom_site_disorder_group  #          9 Check
PLAT860_ALERT_3_G Number of Least-Squares Restraints ............         170 Note
PLAT910_ALERT_3_G Missing # of FCF Reflection(s) Below Theta(Min).          1 Note
PLAT912_ALERT_4_G Missing # of FCF Reflections Above STh/L=  0.600       1704 Note
PLAT933_ALERT_2_G Number of OMIT Records in Embedded .res File ...         21 Note
PLAT941_ALERT_3_G Average HKL Measurement Multiplicity ..........         2.1 Low
PLAT965_ALERT_2_G The SHELXL WEIGHT Optimisation has not Converged     Please Check
PLAT978_ALERT_2_G Number C-C Bonds with Positive Residual Density.          1 Info
```

---

0 **ALERT level A** = Most likely a serious problem - resolve or explain
2 **ALERT level B** = A potentially serious problem, consider carefully
7 **ALERT level C** = Check. Ensure it is not caused by an omission or oversight
78 **ALERT level G** = General information/check it is not something unexpected

1 ALERT type 1 CIF construction/syntax error, inconsistent or missing data

```
19 ALERT type 2 Indicator that the structure model may be wrong or deficient
 4 ALERT type 3 Indicator that the structure quality may be low
62 ALERT type 4 Improvement, methodology, query or suggestion
 1 ALERT type 5 Informative message, check
```

It is advisable to attempt to resolve as many as possible of the alerts in all categories. Often the minor alerts point to easily fixed oversights, errors and omissions in your CIF or refinement strategy, so attention to these fine details can be worthwhile. In order to resolve some of the more serious problems it may be necessary to carry out additional measurements or structure refinements. However, the purpose of your study may justify the reported deviations and the more serious of these should normally be commented upon in the discussion or experimental section of a paper or in the "special_details" fields of the CIF. checkCIF was carefully designed to identify outliers and unusual parameters, but every test has its limitations and alerts that are not important in a particular case may appear. Conversely, the absence of alerts does not guarantee there are no aspects of the results needing attention. It is up to the individual to critically assess their own results and, if necessary, seek expert advice.

Publication of your CIF in IUCr journals

A basic structural check has been run on your CIF. These basic checks will be run on all CIFs submitted for publication in IUCr journals (*Acta Crystallographica*, *Journal of Applied Crystallography*, *Journal of Synchrotron Radiation*); however, if you intend to submit to *Acta Crystallographica Section C* or *E* or *IUCrData*, you should make sure that full publication checks are run on the final version of your CIF prior to submission.

Publication of your CIF in other journals

Please refer to the *Notes for Authors* of the relevant journal for any special instructions relating to CIF submission.

PLATON version of 13/07/2021; check.def file version of 13/07/2021

Datablock tb11 - ellipsoid plot

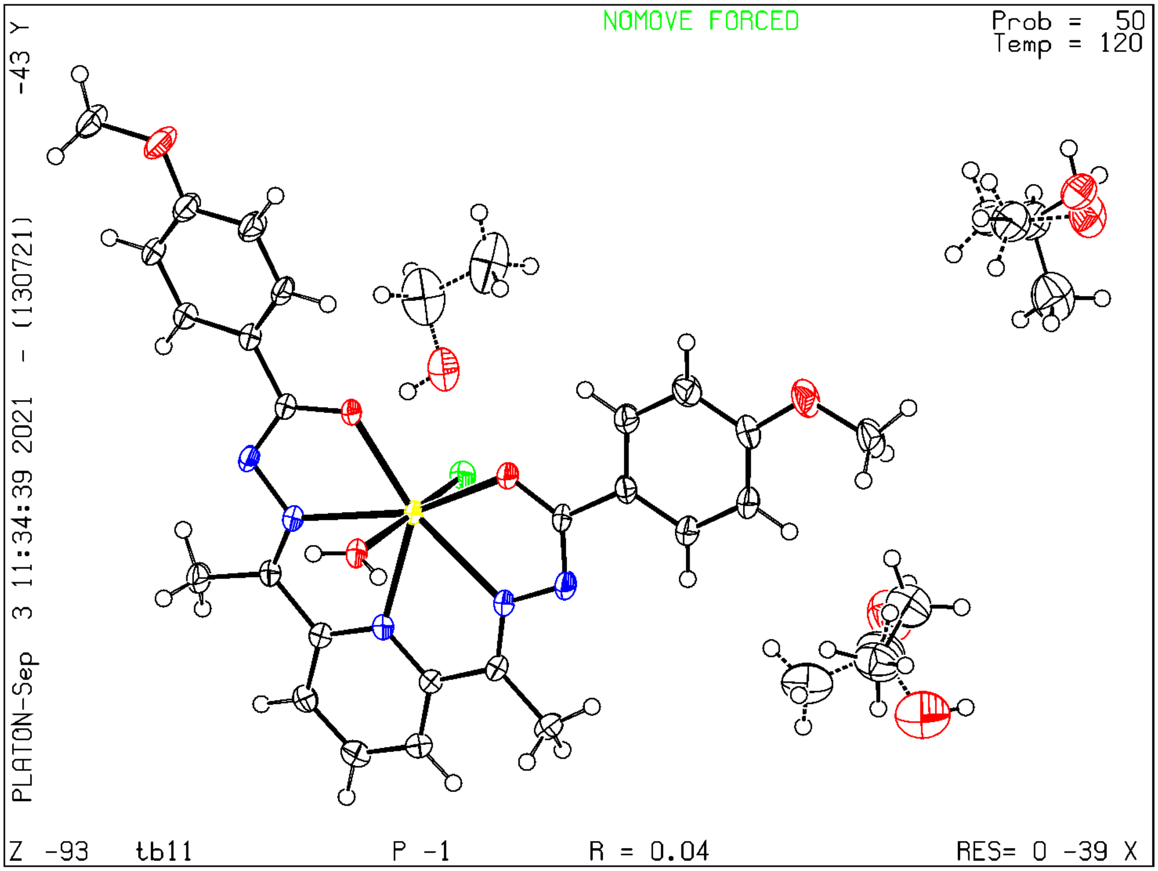

Supplement: Supplementary file 1 [file molecules-26-06908-s001.zip › Supplementary files/2_checkcif.pdf]
