# Supplementary material for: A Series of Novel Pentagonal-Bipyramidal Erbium(III) Complexes with Acyclic Chelating N3O2 Schiff-Base Ligands: Synthesis, Structure, and Magnetism
Source: Molecules. 2021 Nov 16;26(22):6908. doi: 10.3390/molecules26226908 (PMC8622354; doi:10.3390/molecules26226908)

checkCIF/PLATON report

You have not supplied any structure factors. As a result the full set of tests cannot be run.

THIS REPORT IS FOR GUIDANCE ONLY. IF USED AS PART OF A REVIEW PROCEDURE FOR PUBLICATION, IT SHOULD NOT REPLACE THE EXPERTISE OF AN EXPERIENCED CRYSTALLOGRAPHIC REFEREE.

No syntax errors found. CIF dictionary Interpreting this report

Datablock: 6

Bond precision:	C-C = 0.0080 A		Wavelength=0.71073
Cell:	a=18.7925(12)	b=14.9064(8)	c=11.1790(7)
	alpha=90	beta=90	gamma=90
Temperature:	150 K		

	Calculated	Reported
Volume	3131.6(3)	3131.6(3)
Space group	C m c 21	C m c 21
Hall group	C 2c -2	C 2c -2
Moiety formula	C23 H19 Cl2 Er0.05 N5 O4 Y0.96, C6 H16 N	C23 H19 Cl2 Er0.045 N5 O4 Y0.955, C6 H16 N
Sum formula	C29 H35 Cl2 Er0.05 N6 O4 Y0.96	C29 H35 Cl2 Er0.045 N6 O4 Y0.955
Mr	694.97	694.97
Dx,g cm-3	1.474	1.474
Z	4	4
Mu (mm-1)	2.114	2.117
F000	1429.2	1429.0
F000'	1420.13	
h,k,lmax	23,18,13	23,18,13
Nref	3275[1727]	2472
Tmin,Tmax	0.776,0.809	0.727,1.000
Tmin'	0.530	

Correction method= # Reported T Limits: Tmin=0.727 Tmax=1.000
AbsCorr = MULTI-SCAN

Data completeness= 1.43/0.75 Theta(max)= 26.309

R(reflections)= 0.0343(2307)

wR2(reflections)= 0.0730(2472)

S = 1.059

Npar= 212

The following ALERTS were generated. Each ALERT has the format
test-name_ALERT_alert-type_alert-level.
Click on the hyperlinks for more details of the test.

Alert level A

```
PLAT410_ALERT_2_A Short Intra H...H Contact  H2C1     ..H2C1     .       1.61 Ang.
                                                -2-x,y,z  =      4_355 Check
```

Alert level B

```
PLAT214_ALERT_2_B Atom C2C       (Anion/Solvent) ADP max/min Ratio        5.6 prolat
```

Alert level C

```
PLAT041_ALERT_1_C Calc. and Reported SumFormula    Strings  Differ     Please Check
PLAT077_ALERT_4_C Unitcell Contains Non-integer Number of Atoms ..     Please Check
PLAT221_ALERT_2_C Solv./Anion  Resd 2  C   Ueq(max)/Ueq(min) Range        5.1 Ratio
PLAT223_ALERT_4_C Solv./Anion  Resd 2  H   Ueq(max)/Ueq(min) Range       10.0 Ratio
PLAT243_ALERT_4_C High   'Solvent' Ueq as Compared to Neighbors of        C2C Check
PLAT244_ALERT_4_C Low    'Solvent' Ueq as Compared to Neighbors of        N1C Check
PLAT245_ALERT_2_C U(iso) H1N       Smaller than U(eq) N1C       by      0.021 Ang**2
PLAT360_ALERT_2_C Short  C(sp3)-C(sp3) Bond  C1C      - C2C      .       1.34 Ang.
PLAT420_ALERT_2_C D-H Bond Without Acceptor  N1C      --H1N      .     Please Check
PLAT790_ALERT_4_C Centre of Gravity not Within Unit Cell: Resd.  #          1 Note
              C23 H19 Cl2 Er0.05 N5 O4 Y0.96
```

Alert level G

```
PLAT007_ALERT_5_G Number of Unrefined Donor-H Atoms .............          1 Report
PLAT042_ALERT_1_G Calc. and Reported Moiety Formula Strings Differ     Please Check
PLAT068_ALERT_1_G Reported F000 Differs from Calcd (or Missing)...     Please Check
PLAT168_ALERT_4_G The CIF-Embedded .res File Contains EXYZ Records          1 Report
PLAT171_ALERT_4_G The CIF-Embedded .res File Contains EADP Records          1 Report
PLAT300_ALERT_4_G Atom Site Occupancy of Er1        Constrained at      0.045 Check
PLAT300_ALERT_4_G Atom Site Occupancy of Y1         Constrained at      0.955 Check
PLAT300_ALERT_4_G Atom Site Occupancy of C3C        Constrained at        0.5 Check
PLAT300_ALERT_4_G Atom Site Occupancy of C4C        Constrained at        0.5 Check
PLAT300_ALERT_4_G Atom Site Occupancy of H4C1       Constrained at        0.5 Check
PLAT300_ALERT_4_G Atom Site Occupancy of H4C2       Constrained at        0.5 Check
PLAT300_ALERT_4_G Atom Site Occupancy of H4C3       Constrained at        0.5 Check
PLAT300_ALERT_4_G Atom Site Occupancy of H3C1       Constrained at        0.5 Check
PLAT300_ALERT_4_G Atom Site Occupancy of H3C2       Constrained at        0.5 Check
PLAT301_ALERT_3_G Main Residue  Disorder ..............(Resd  1  )         6% Note
PLAT302_ALERT_4_G Anion/Solvent/Minor-Residue Disorder (Resd  2  )        29% Note
PLAT344_ALERT_2_G Unusual sp3       Angle Range in Solvent/Ion for        C2C Check
PLAT380_ALERT_4_G Incorrectly? Oriented X(sp2)-Methyl Moiety .....        C15 Check
PLAT410_ALERT_2_G Short Intra H...H Contact  H3C1     ..H2C2     .       1.82 Ang.
                                                  x,y,z  =      1_555 Check
PLAT410_ALERT_2_G Short Intra H...H Contact  H3C2     ..H2C2     .       1.80 Ang.
```

```
                                                  x,y,z  =      1_555 Check
PLAT720_ALERT_4_G Number of Unusual/Non-Standard Labels .........         11 Note
PLAT789_ALERT_4_G Atoms with Negative _atom_site_disorder_group  #          7 Check
PLAT790_ALERT_4_G Centre of Gravity not Within Unit Cell: Resd.  #          2 Note
              C6 H16 N
PLAT802_ALERT_4_G CIF Input Record(s) with more than 80 Characters          1 Info
PLAT941_ALERT_3_G Average HKL Measurement Multiplicity ..........         2.5 Low
PLAT965_ALERT_2_G The SHELXL WEIGHT Optimisation has not Converged     Please Check
```

```
   1 ALERT level A = Most likely a serious problem - resolve or explain
   1 ALERT level B = A potentially serious problem, consider carefully
  10 ALERT level C = Check. Ensure it is not caused by an omission or oversight
  26 ALERT level G = General information/check it is not something unexpected

   3 ALERT type 1 CIF construction/syntax error, inconsistent or missing data
  10 ALERT type 2 Indicator that the structure model may be wrong or deficient
   2 ALERT type 3 Indicator that the structure quality may be low
  22 ALERT type 4 Improvement, methodology, query or suggestion
   1 ALERT type 5 Informative message, check
```

It is advisable to attempt to resolve as many as possible of the alerts in all categories. Often the minor alerts point to easily fixed oversights, errors and omissions in your CIF or refinement strategy, so attention to these fine details can be worthwhile. In order to resolve some of the more serious problems it may be necessary to carry out additional measurements or structure refinements. However, the purpose of your study may justify the reported deviations and the more serious of these should normally be commented upon in the discussion or experimental section of a paper or in the "special_details" fields of the CIF. checkCIF was carefully designed to identify outliers and unusual parameters, but every test has its limitations and alerts that are not important in a particular case may appear. Conversely, the absence of alerts does not guarantee there are no aspects of the results needing attention. It is up to the individual to critically assess their own results and, if necessary, seek expert advice.

Publication of your CIF in IUCr journals

A basic structural check has been run on your CIF. These basic checks will be run on all CIFs submitted for publication in IUCr journals (*Acta Crystallographica*, *Journal of Applied Crystallography*, *Journal of Synchrotron Radiation*); however, if you intend to submit to *Acta Crystallographica Section C* or *E* or *IUCrData*, you should make sure that full publication checks are run on the final version of your CIF prior to submission.

Publication of your CIF in other journals

Please refer to the *Notes for Authors* of the relevant journal for any special instructions relating to CIF submission.

PLATON version of 13/07/2021; check.def file version of 13/07/2021

Datablock 6 - ellipsoid plot

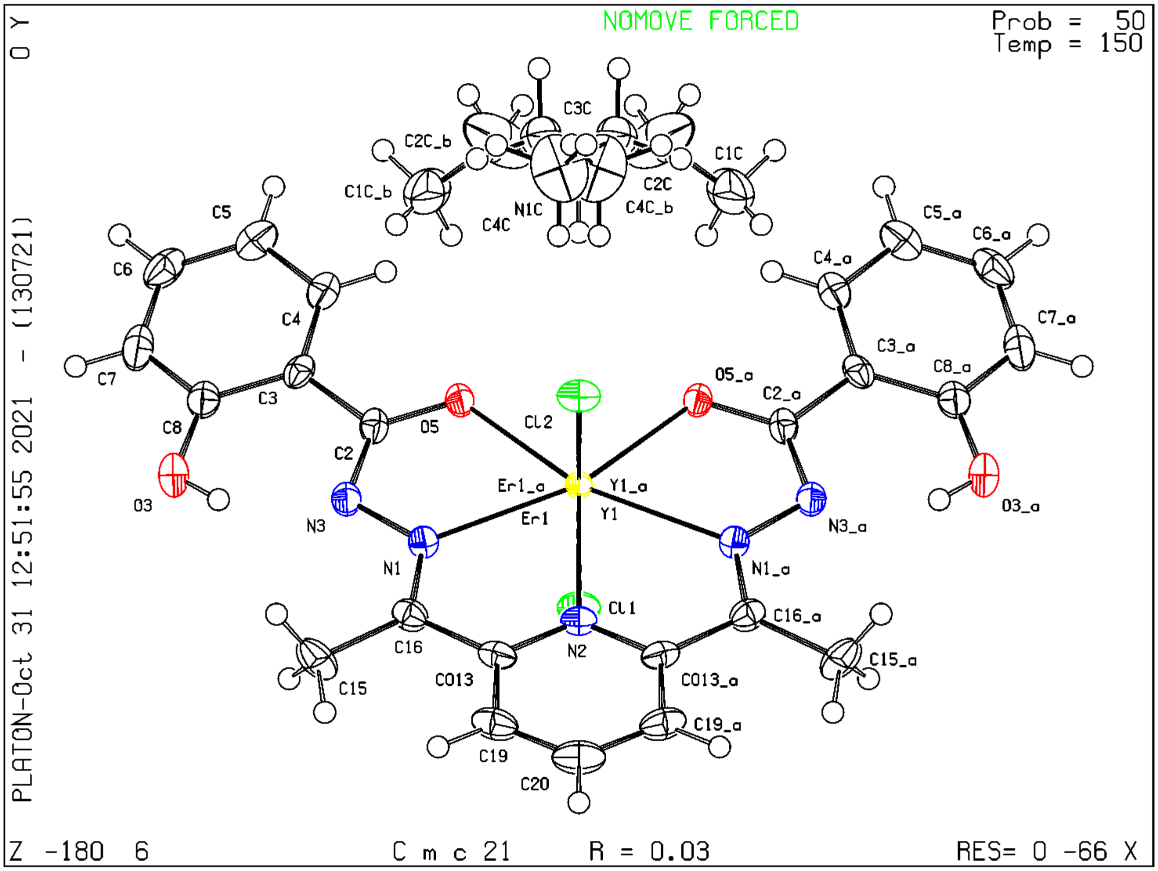

Supplement: Supplementary file 1 [file molecules-26-06908-s001.zip › Supplementary files/6_checkcif.pdf]
