# Supplementary material for: A Series of Novel Pentagonal-Bipyramidal Erbium(III) Complexes with Acyclic Chelating N3O2 Schiff-Base Ligands: Synthesis, Structure, and Magnetism
Source: Molecules. 2021 Nov 16;26(22):6908. doi: 10.3390/molecules26226908 (PMC8622354; doi:10.3390/molecules26226908)
Supplement: Supplementary file 1 [file molecules-26-06908-s001.zip › Supplementary files/ESI Molecules corrected.pdf]

Electronic Supplementary Information

A series of novel pentagonal-bipyramidal erbium(III) complexes with acyclic chelating N_3O_2 Schiff-base ligands: synthesis, structure and magnetism

Tamara A. Bazhenova [1,*], Vyacheslav A. Kopotkov [1,*], Denis V. Korchagin [1], Yuriy V. Manakin [1], Leokadiya V. Zorina [1,2], Sergey V. Simonov [1,2], Ilya A.Yakushev [1,3], Vladimir S. Mironov [1,4,*], Alexander N. Vasiliev [5,6], Olga V. Maximova[6] and Eduard B.Yagubskii[1,*]

1 Institute of Problems of Chemical Physics, IPCP RAS, Chernogolovka 142432, Russia; tabazh@mail.ru (T.A.B.); slavaoven@mail.ru (V.A.K.); yagubski@gmail.com (E.B.Y);
2 Institute of Solid State Physics, ISSP RAS, Chernogolovka 142432, Russia;
3 Kurnakov Institute of General and Inorganic Chemistry, IGIC RAS, Moscow, Russia;
4 Shubnikov Institute of Crystallography of Federal Scientific Research Centre "Crystallography and Photonics" RAS, Moscow, Russia; mirsa@list.ru (V.S.M.);
5 National University of Science and Technology "MISiS", Moscow 119049, Russia;
6 Lomonosov Moscow State University, Moscow119991, Russia.
* Correspondence: slavaoven@mail.ru (V.A.K.), mirsa@list.ru (V.S.M.), yagubski@gmail.com (E.B.Y)

Table S1. *SHAPE*[*] analysis

Complex	Polyhedra						
	HP	HPY	**PBPY**	COC	CTPR	JPBPY	JETPY
$[Er(DAPMBH)(H_2O)Cl]\cdot 2C_2H_5OH$ **2**	33.059	22.894	**1.171**	8.012	6.564	5.597	22.339
$[Er(DAPMBH)(CH_3OH)Cl]$ **3**	33.569	23.744	**1.457**	8.209	6.289	6.018	21.679
$[Er(DAPMBH)(CH_3OH)(N_3)]$ **4**	32.449	20.571	**1.365**	7.198	5.811	4.527	19.949
$(HNEt_3)[Er(H_2DAPS)Cl_2]$ **5**	33.747	25.236	**1.031**	9.050	7.276	6.505	24.543
$(HNEt_3)[Er_{0.05}Y_{0.95}(H_2DAPS)Cl_2]$ **6**	33.532	24.971	**1.136**	9.038	7.293	6.618	24.327

HP – Heptagon (D_{7h}), HPY – Hexagonal pyramid (C_{6v}), **PBPY – Pentagonal bipyramid (D_{5h})**, COC – Capped octahedron (C_{3v}), CTPR – Capped trigonal prism (C_{2v}), JPBPY – Johnson pentagonal bipyramid (D_{5h}), JETPY – Johnson elongated triangular pyramid (C_{3v}).

* [M. Llunell, D. Casanova, J. Cirera, P. Alemany, S. Alvarez, 'SHAPE: Program for the Stereochemical Analysis of Molecular Fragments by Means of Continuous Shape Measures and Associated Tools', Version 2.1, 2013, Barcelona]

Er(DAPMBH)(H_2O)Cl]·2EtOH (2)

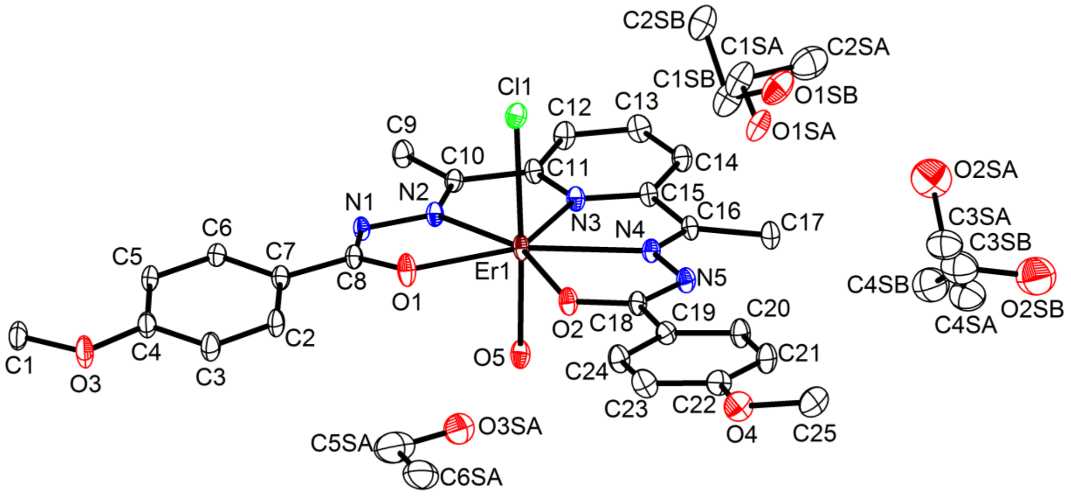

Figure S1. Asymmetric unit with atom numbering scheme in **2** (30% thermal ellipsoids, H atoms are omitted for clarity). Occupancy of disordered EtOH solvent molecules: O1Sa – 0.8, O1Sb – 0.2, O2Sa – 0.6, O2Sb - 0.2, O3Sa – 0.2.

Table S2. Selected bond lengths (Å) and angles (º) in **2**.

Er1-O1	2.218(3)	O1-C8	1.292(5)	O2-C18	1.295(5)
Er1-O2	2.244(3)	C8-N1	1.328(6)	C18-N5	1.319(6)
Er1-N2	2.429(3)	N1-N2	1.394(5)	N5-N4	1.378(5)
Er1-N4	2.404(4)	N2-C10	1.291(6)	N4-C16	1.291(6)
Er1-N3	2.404(4)	C10-C11	1.494(6)	C16-C15	1.478(6)
Er1-O5	2.275(3)	C11-N3	1.348(5)	C15-N3	1.353(5)
Er1-Cl1	2.6087(12)				

O1-Er1-N2	65.89(12)	O2-Er1-N4	66.20(12)
O1-Er1-N3	131.86(12)	O2-Er1-N3	132.15(12)
O1-Er1-N4	161.18(13)	O2-Er1-N2	160.22(12)
N2-Er1-N3	66.00(12)	N4-Er1-N3	66.07(12)
O1-Er1-O2	95.38(12)	N2-Er1-N4	131.80(12)
O1-Er1-O5	87.17(13)	O2-Er1-O5	91.32(12)
N2-Er1-O5	81.84(12)	N4-Er1-O5	89.32(12)
O1-Er1-Cl1	93.06(10)	O2-Er1-Cl1	97.57(9)
N2-Er1-Cl1	90.08(9)	N4-Er1-Cl1	93.25(9)
N3-Er1-O5	84.95(13)	N3-Er1-Cl1	88.26(9)
O5-Er1-Cl1	171.05(9)		

The shortest Er…Er distances in **2** are found in the *ab* plane (Fig. S2a). Two Er complexes joined by a pair of equivalent hydrogen O(5)$_{H2O}$-H…N(1)$_{DAPMBH}$ bonds (H…N of 1.98(4) Å) form a dimer with Er…Er separation of 7.0386(4) Å (Fig. S2b). The dimers are connected along *a*-axis by C(17)$_{DAPMBH}$-H…Cl(1) contact and hydrogen bonding through solvent bridges: O(5)$_{H2O}$-H…O(1Sa)$_{ethanol}$-H…Cl(1) or O(5)$_{H2O}$-H…O(1Sb)$_{ethanol}$-H…O(1)$_{DAPMBH}$ (hydrogen bonds geometry is given in Table S3), Er…Er separation is 8.3532(4) Å. Third shortest Er…Er distance in the *ab* plane is 8.5853(4) Å between the complexes connected by C(17)$_{DAPMBH}$-H…Cl(1) hydrogen contacts (Table S3). All the DAPMBH average planes in the *ab*-layer are parallel and a number of C…C shortened contacts less than the sum of van der Waals radii (3.6

Å) are also formed between their π-stacked fragments. The *ab* layers of the Er complexes are separated by ethanol solvent molecules along *c*. The ethanol molecules occupy partially filled positions in large channels running along *a*-axis and they can leave the crystal quite easily after evaporation of the mother liquid.

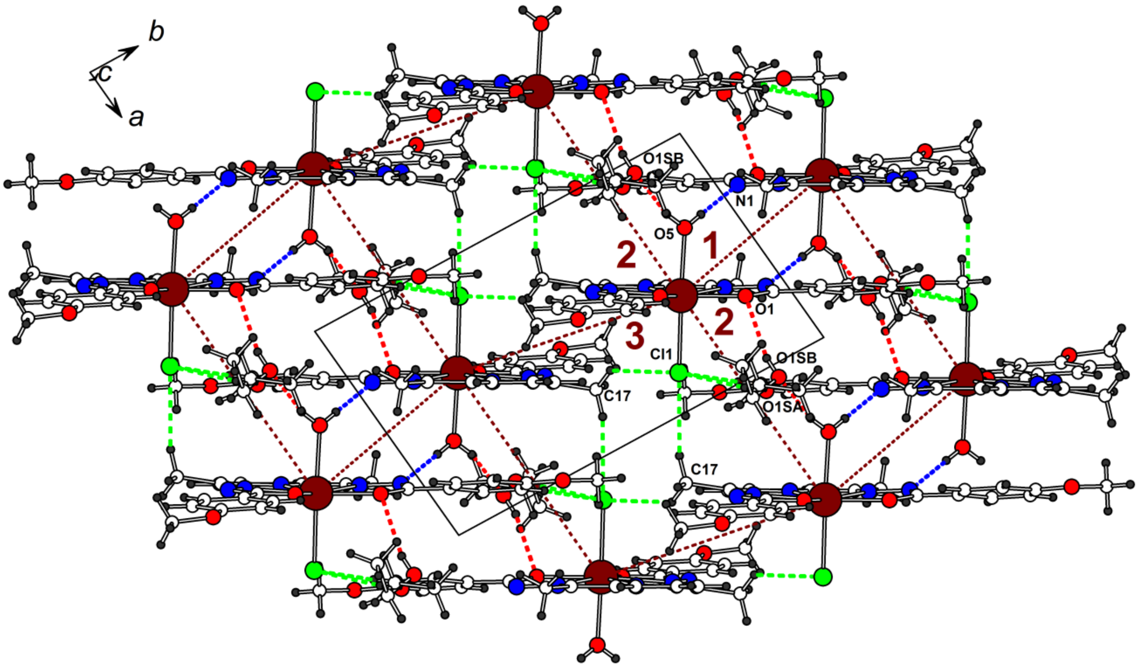

(a)

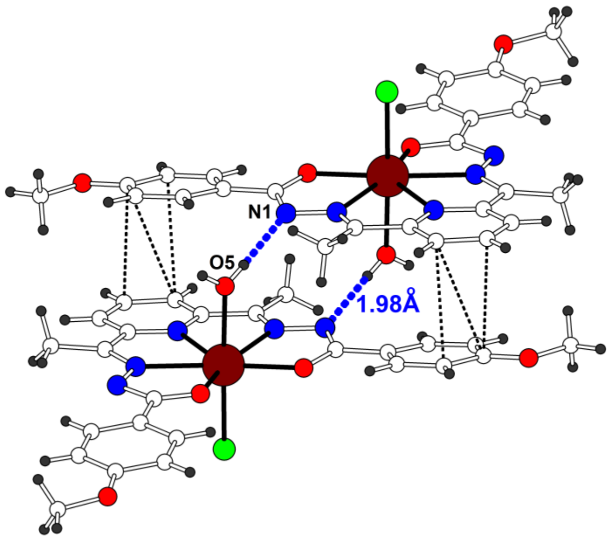

(b)

Figure S2. (a) The *ab* layer of Er complexes in **2**. O-H…N, O/C-H…O, O/C-H…Cl contacts are shown by blue, red and green dashed lines, respectively. The shortest Er…Er separations (brown dotted lines) are 7.0386(4) Å (1, dimer), 8.3532(4) Å (2) and 8.5853(4) Å (3). (b) Centrosymmetric H-bonded dimer in **2**. C…C contacts < 3.6 Å are shown by black dotted lines.

Table S3. Hydrogen bond geometry in **2**.

D	H	A	Symmetry code for A	D-H, Å	H…A, Å	D…A, Å	D-H…A, º
O5	H5b	N1	1-x, 2-y, -z	0.82(4)	1.98(4)	2.795(5)	174(6)
C5	H5	Cl1	2-x, 2-y, -z	0.95	2.91	3.849(4)	170
C6	H6	O5	1-x, 2-y, -z	0.95	2.60	3.232(6)	124
C13	H13	O4	x, y, z-1	0.95	2.60	3.436(6)	147
C17	H17b	Cl1	x-1, y, z	0.98	2.85	3.627(5)	136
C17	H17c	Cl1	x-1, y, z	0.98	2.83	3.714(5)	150
C25	H25b	Cl1	1-x,1-y,1-z	0.98	2.79	3.728(6)	161
O1Sa	H1Sa	Cl1	x-1, y, z	0.84	2.39	3.224(6)	169
O1Sb	H1Sb	O1	x-1, y, z	0.84	2.26	3.06(3)	161
O5	H5a	O1Sa	x, y, z	0.82(4)	1.81(4)	2.622(6)	172(6)
O5	H5a	O1Sb	x, y, z	0.82(4)	2.02(4)	2.82(2)	164(6)
C12	H12	O2Sa	1-x, 1-y, -z	0.95	2.29	3.212(10)	163

[Er(DAPMBH)(MeOH)Cl] (3)

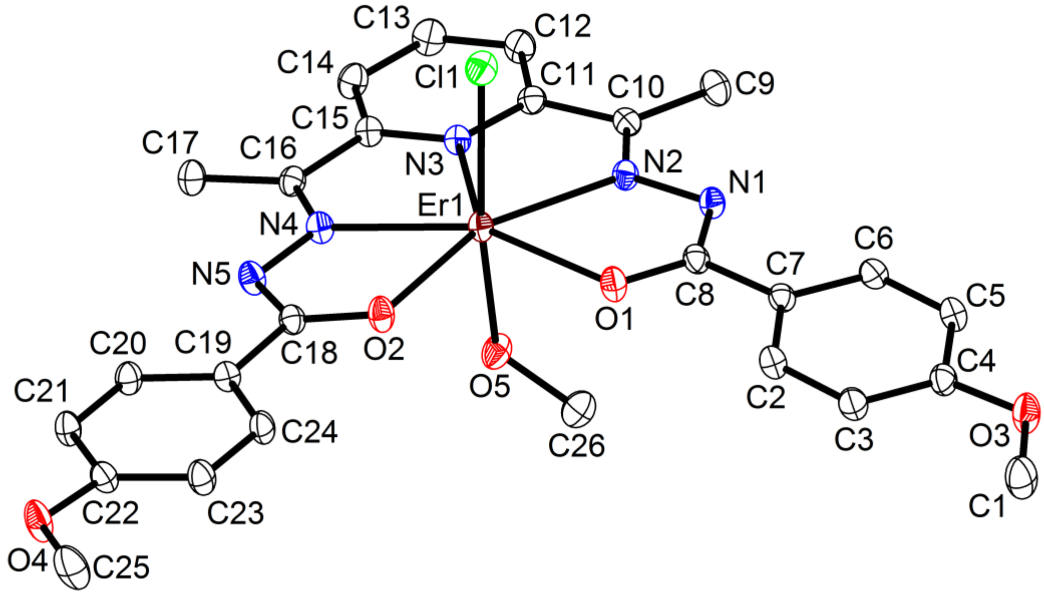

Figure S3. Asymmetric unit with atom numbering scheme in **3** (50% thermal ellipsoids, H atoms are omitted for clarity).

Table S4. Selected bond lengths (Å) and angles (º) in **3**.

Er1-O1	2.2438(15)	O1-C8	1.297(2)	O2-C18	1.295(2)
Er1-O2	2.2422(15)	C8-N1	1.327(3)	C18-N5	1.322(3)
Er1-N2	2.4180(17)	N1-N2	1.389(2)	N5-N4	1.381(2)
Er1-N4	2.4014(17)	N2-C10	1.296(3)	N4-C16	1.294(3)
Er1-N3	2.4312(17)	C10-C11	1.482(3)	C16-C15	1.480(3)
Er1-O5	2.3285(16)	C11-N3	1.348(3)	C15-N3	1.352(3)
Er1-Cl1	2.6009(5)				

O1-Er1-N2	66.31(6)	O2-Er1-N4	66.54(6)
O1-Er1-N3	130.78(5)	O2-Er1-N3	131.81(5)
O1-Er1-N4	157.51(6)	O2-Er1-N2	159.91(6)
N2-Er1-N3	65.05(6)	N4-Er1-N3	65.36(6)
O1-Er1-O2	94.91(5)	N2-Er1-N4	129.59(6)
O1-Er1-O5	85.53(6)	O2-Er1-O5	90.43(6)
N2-Er1-O5	81.39(6)	N4-Er1-O5	82.16(6)
O1-Er1-Cl1	94.96(4)	O2-Er1-Cl1	100.60(4)
N2-Er1-Cl1	88.61(4)	N4-Er1-Cl1	100.73(4)
N3-Er1-O5	80.41(6)	N3-Er1-Cl1	90.98(4)
O5-Er1-Cl1	168.87(5)		

The Er complexes in **3** form infinite chains along *a* (Fig. S4a) with Er…Er distances of 7.0338(2) Å and hydrogen bonding of O-H…Cl type between MeOH and Cl ligands of adjacent Er units (interaction **1** in Fig. S4b). The H…Cl distance in this bond is as short as 2.33(4) Å (see Table S5 for hydrogen bond geometry). The chains are joined in the *ac* plane by C-H…Cl, C-H…O hydrogen bonds and π-stacking of DAPMBH ligands (Fig. S4b). There are 14 C…C

contacts less than sum of van der Waals radii in the interaction **2** with Er…Er separation of 7.6231(5) Å and only six such contacts on the other side of the DAPMBH ligand.

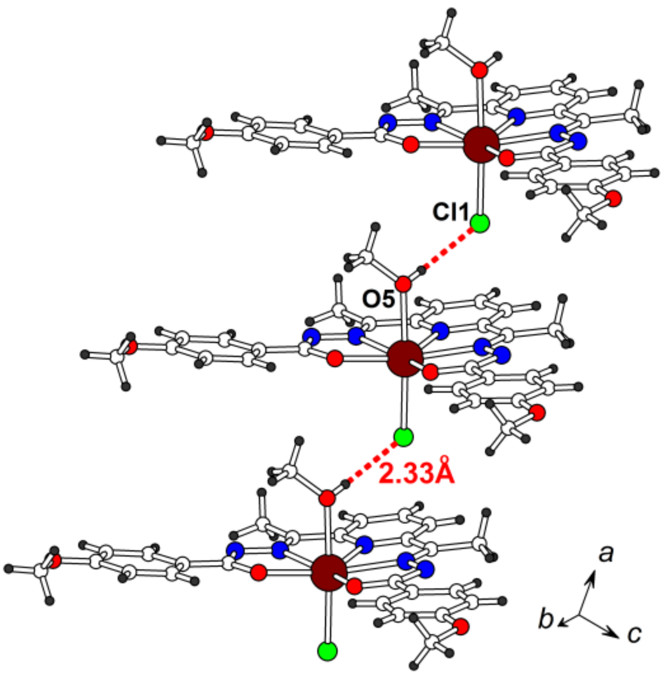

(a)

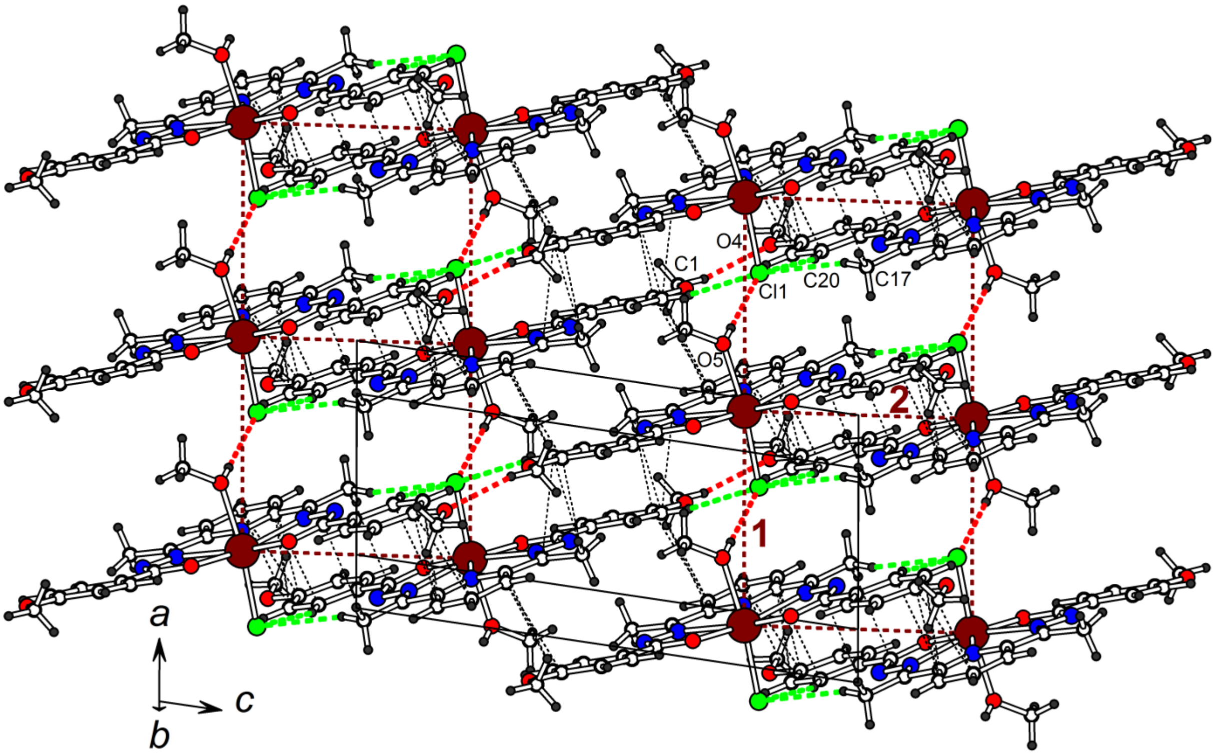

(b)

Figure S4. (a) Infinite chain of hydrogen bonded Er complexes in **3**. (b)View of the *ac* layer in the structure **3**. Hydrogen bonds (red dashed lines for C-H…O and O-H…Cl, green dashed lines for C-H…Cl), Er…Er distances (brown dashed lines, 1 = 7.0338(2) Å, 2 = 7.6231(5) Å), C…C contacts < 3.6 Å (black dotted lines) are shown.

Table S5. Hydrogen bond geometry in **3**.

D	H	A	Symmetry code for A	D-H, Å	H…A, Å	D…A, Å	D-H…A, º
O5	H5a	Cl1	x-1, y, z	0.73(3)	2.33(4)	3.0523(17)	172(4)
C1	H1b	O4	1+x, y, 1+z	0.98	2.45	3.358(3)	154
C17	H17c	Cl1	2-x, -y, 2-z	0.98	2.95	3.842(2)	152
C20	H20	Cl1	2-x, -y, 2-z	0.95	2.84	3.718(2)	154

[Er(DAPMBH)(CH_3OH)(N_3)] (4)

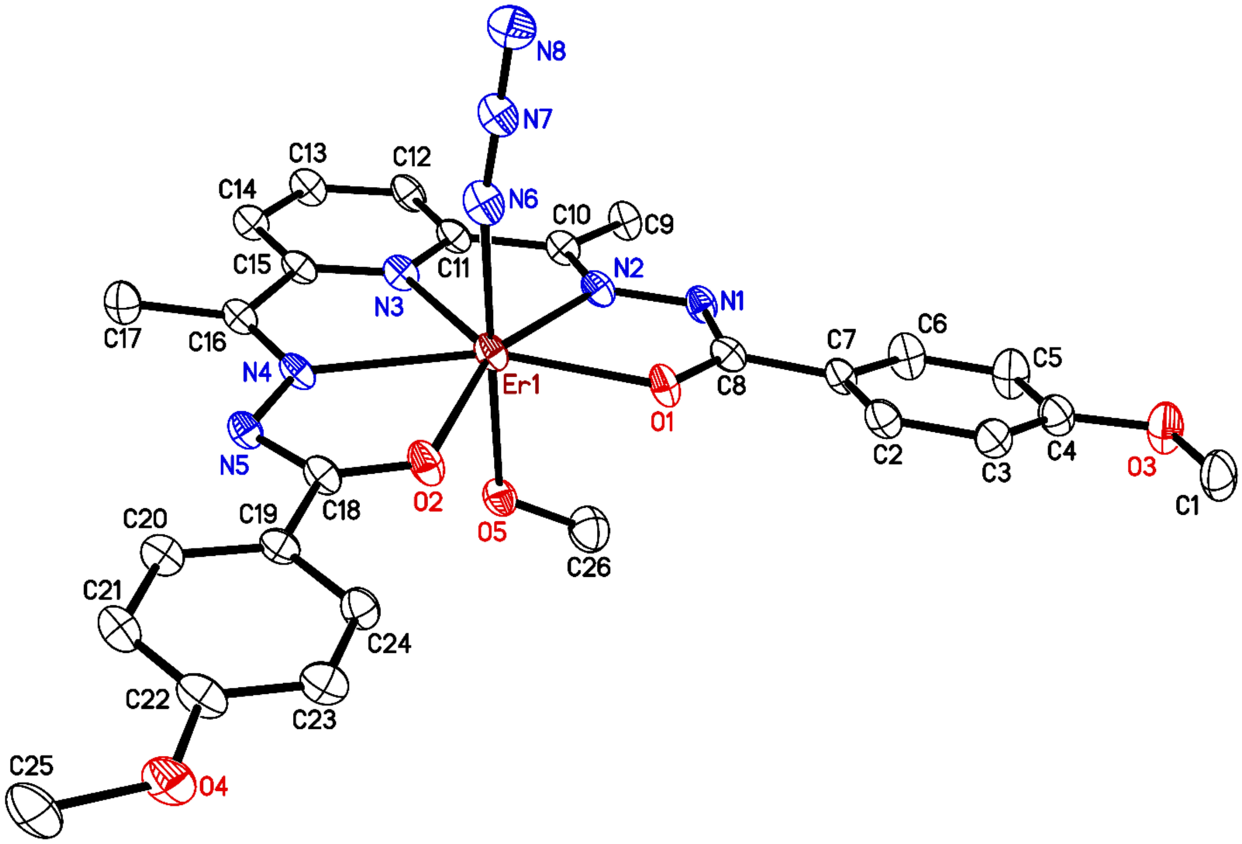

Figure S5. Asymmetric unit with atom numbering scheme in **[Er(DAPMBH)(CH_3OH)(N_3)] (4)** (35% thermal ellipsoids, H atoms are omitted for clarity).

Table S6. Selected bond lengths (Å) and angles (º) in **4**.

Er1-O1	2.248(5)	O1-C8	1.298(9)	O2-C18	1.268(9)
Er1-O2	2.278(5)	C8-N1	1.326(9)	C18-N5	1.349(10)
Er1-N2	2.407(6)	N1-N2	1.379(9)	N5-N4	1.396(8)
Er1-N4	2.452(6)	N2-C10	1.281(10)	N4-C16	1.292(9)
Er1-N3	2.436(6)	C10-C11	1.487(11)	C16-C15	1.488(9)
Er1-O5	2.316(5)	C11-N3	1.346(9)	C15-N3	1.337(10)
Er1-N6	2.232(7)	N6-N7	1.187(10)	N7-N8	1.152(10)

O1-Er1-N2	66.6(2)	O2-Er1-N4	65.25(19)
O1-Er1-N3	131.94(19)	O2-Er1-N3	129.7(2)
O1-Er1-N4	160.85(19)	O2-Er1-N2	160.4(2)
N2-Er1-N3	65.4(2)	N4-Er1-N3	64.7(2)
O1-Er1-O2	96.72(19)	N2-Er1-N4	64.7(2)
O1-Er1-O5	83.5(2)	O2-Er1-O5	87.3(2)
N2-Er1-O5	80.8(2)	N4-Er1-O5	89.0(2)
O1-Er1-N6	96.0(2)	O2-Er1-N6	92.9(2)
N2-Er1-N6	98.9(2)	N4-Er1-N6	91.5(2)
N3-Er1-O5	86.7(2)	N3-Er1-N6	93.6(2)
O5-Er1-N6	179.5(2)	N8-N7-N6	179.4(9)

The Er(III) complexes with **DAPMBH** equatorial ligand in **4** form hydrogen-bonded centrosymmetric dimers (Fig. S6) with Er…Er intradimer separation of 6.6939(17) Å. In turn,

each of these units are shown short contacts between neighboring complex molecules (Fig. S7), thus forming one-dimensional polymeric chains along *a* axis of the unit cell (Fig. S8). The nearest Er…Er distance outside dimeric units is 7.5588(16) Å. Hydrogen bond geometry is given in Table S7.

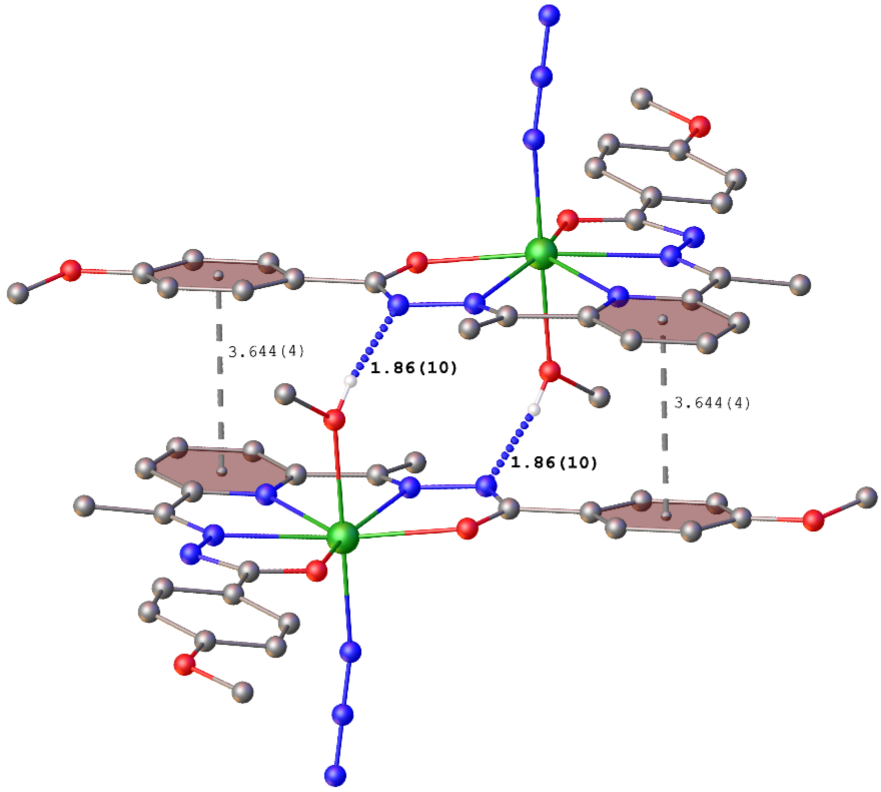

Figure S6. Dimeric hydrogen-bonded units in crystal structure of **[Er(DAPMBH)(CH_3OH)N_3] (4)**. The hydrogen bond O-H…N are shown with blue dotted lines, π-π stacking interaction between aromatic systems of the ligands are shown with grey dashed lines. Color code: erbium – green, oxygen – red, nitrogen – blue, carbon – grey. All distances are given in Å.

Table S7. Hydrogen bond geometry in **4**.

D-H...A	d(D-H)	d(H...A)	d(D...A)	<(DHA)
O(5)-H(5)...N(5)#1	0.89(10)	1.80(10)	2.688(8)	173(9)

Symmetry transformations used to generate equivalent atoms:
#1 -x+1,-y+1,-z+1

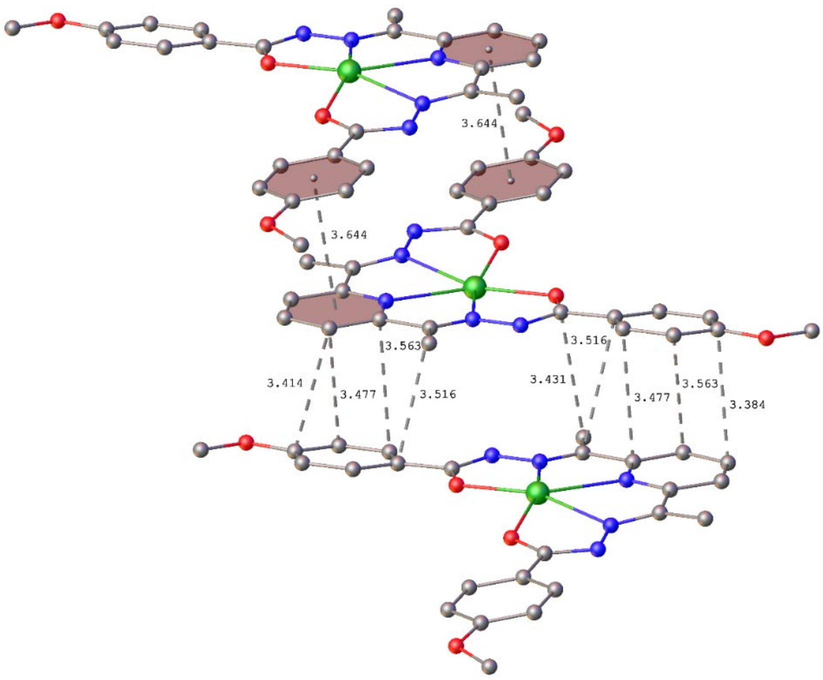

Figure S7. Short intermolecular contacts in crystal structure packing of **[Er(DAPMBH)(CH_3OH)N_3]**. In addition to π-π stacking interaction short contacts between carbon atoms are shown (C…C < 3.6 Å, all distances are given in Å). Azide anions and methanol molecules are omitted for clarity.

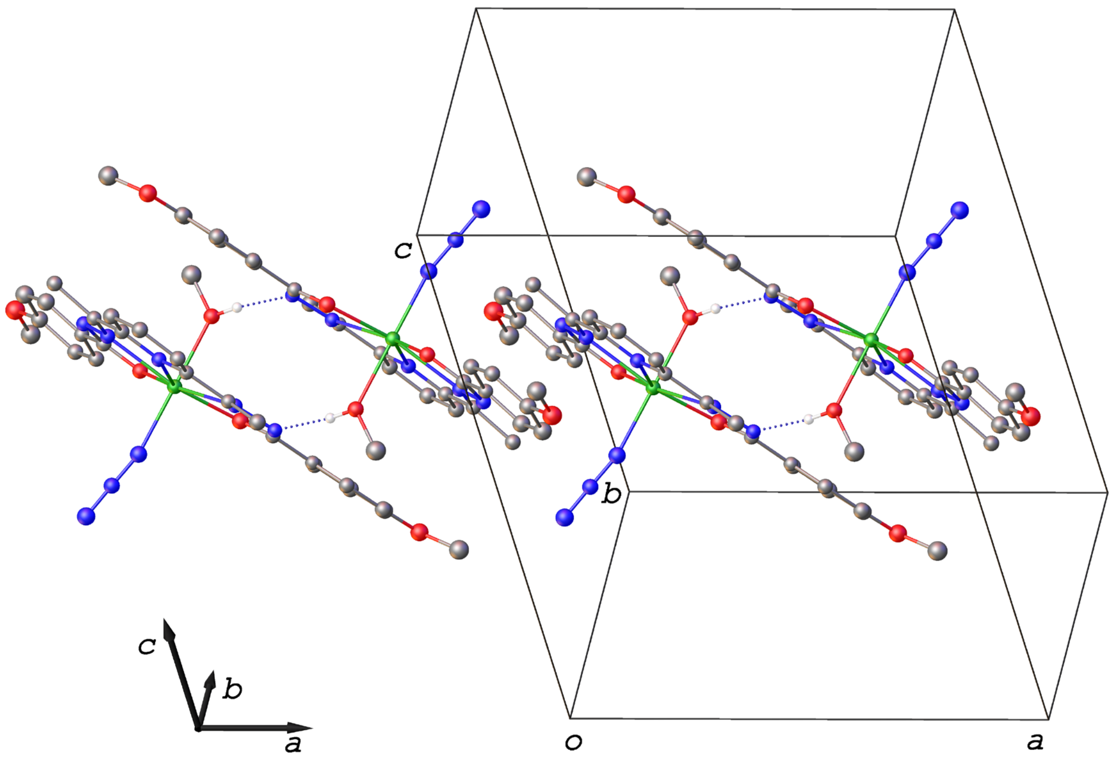

Figure S8. Fragment of *1D* polymeric chain of the complex **4**, mutual arrangement of two doubly hydrogen bonded units are shown. Most of the hydrogen atoms are omitted for clarity.

[Er(H_2DAPS)Cl_2]$^-$[(Et_3H)N]$^+$ (5) and [$Y_{0.95}Er_{0.05}$(H_2DAPS)Cl_2]$^-$[(Et_3H)N]$^+$ (6)

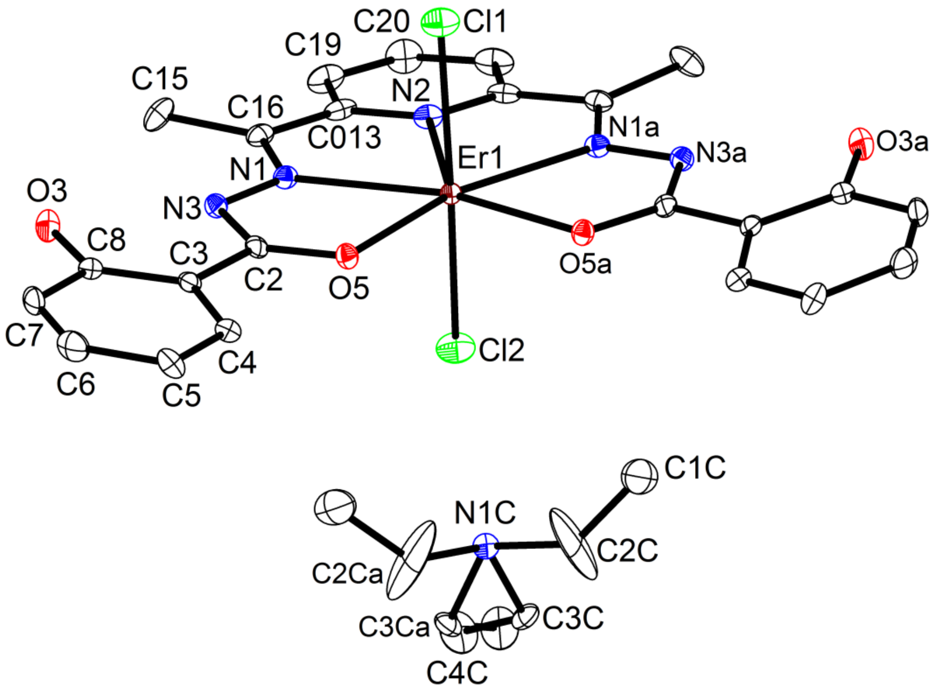

Figure S9. Asymmetric unit with atom numbering scheme in **5** (30% thermal ellipsoids, H atoms are omitted for clarity).

Table S8. Selected bond lengths (Å) and angles (º) in **5** and **6**.

Er(1)-O(5)	2.246(3)	Y(1)-O(5)	2.256(3)
Er(1)-O(5a)	2.246(3)	Y(1)-O(5a)	2.256(3)
Er(1)-N(1)	2.425(4)	Y(1)-N(1)	2.436(3)
Er(1)-N(1a)	2.424(4)	Y(1)-N(1a)	2.436(3)
Er(1)-N(2)	2.424(6)	Y(1)-N(2)	2.428(4)
Er(1)-Cl(1)	2.5862(16)	Y(1)-Cl(1)	2.5990(15)
Er(1)-Cl(2)	2.6543(17)	Y(1)-Cl(2)	2.6607(15)
O(5a)-Er(1)-O(5)	96.00(17)	O(5)-Y(1)-O(5a)	97.59(13)
O(5)-Er(1)-N(2)	131.97(8)	O(5)-Y(1)-N(2)	131.19(6)
O(5)-Er(1)-N(1)	66.45(13)	O(5)-Y(1)-N(1)	65.79(10)
O(5)-Er(1)-N(1a)	162.43(12)	O(5)-Y(1)-N(1a)	163.37(10)
N(1)-Er(1)-N(2)	65.54(9)	N(2)-Y(1)-N(1)	65.41(7)
O(5)-Er(1)-Cl(1)	94.81(9)	O(5)-Y(1)-Cl(1)	94.48(7)
N(1a)-Er(1)-N(1)	131.09(19)	N(1)-Y(1)-N(1a)	130.83(14)
N(1a)-Er(1)-N(2)	65.54(9)	N(2)-Y(1)-N(1a)	65.41(7)
N(2)-Er(1)-Cl(1)	84.70(14)	N(2)-Y(1)-Cl(1)	84.74(12)
N(1)-Er(1)-Cl(1)	87.76(9)	N(1)-Y(1)-Cl(1)	87.63(8)
O(5)-Er(1)-Cl(2)	94.42(8)	O(5)-Y(1)-Cl(2)	94.57(7)
N(2)-Er(1)-Cl(2)	81.49(14)	N(2)-Y(1)-Cl(2)	81.50(12)
N(1)-Er(1)-Cl(2)	86.54(9)	N(1)-Y(1)-Cl(2)	86.66(8)
Cl(1)-Er(1)-Cl(2)	166.19(5)	Cl(1)-Y(1)-Cl(2)	166.24(4)

Symmetry transformations used to generate equivalent atoms: (a) -x-2,y,z

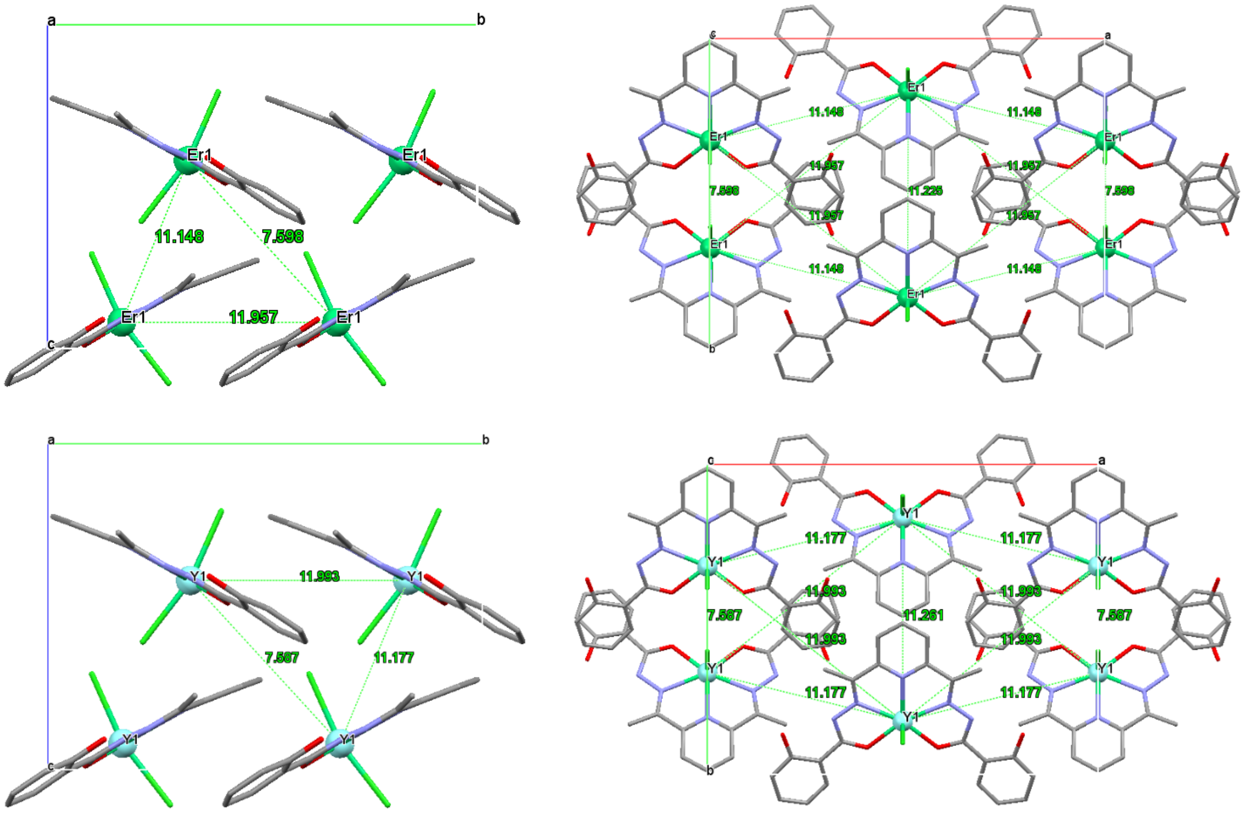

Figure S10. Unit cell contents in the crystal packing of **5** and **6**. along crystallographic *a* (left) and *c* (right) axes. The inter Er-Er and Y-Y distances of the neighbour molecules are shown by green dashed lines (values are in Å). The Et_3NH counter cations and H atoms are omitted for clarity.

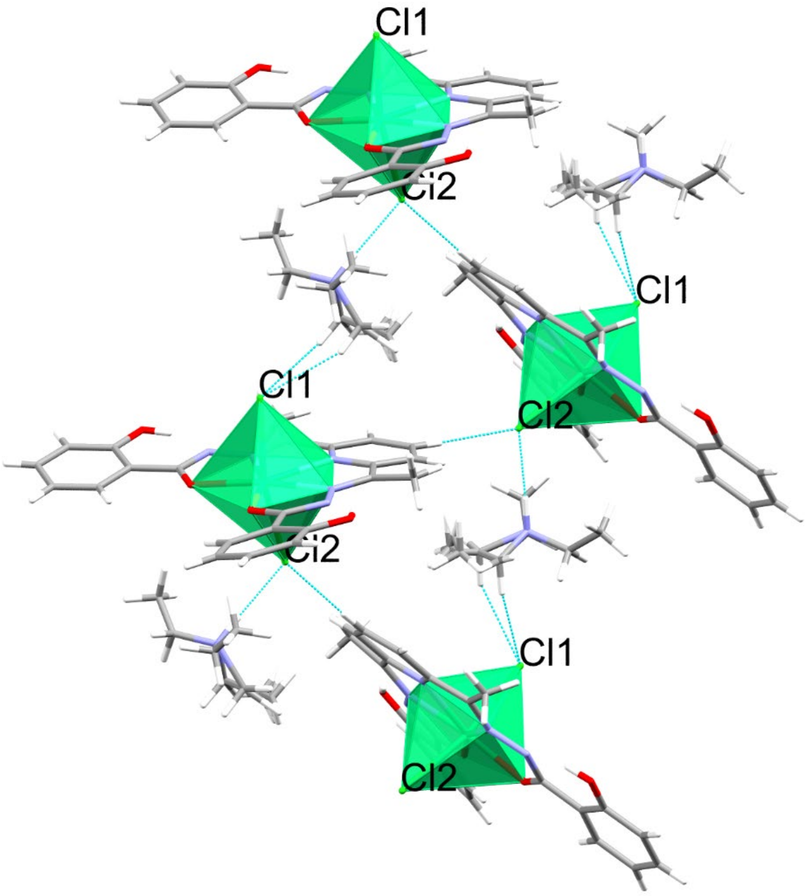

Figure S11. Fragment of the crystal packing of **5**. Dotted cyan lines show the intermolecular contacts.

Table S9. Comparison of the bond length values (Å) in Er (or Y in **6**) polyhedron in the structures **2-6**

	**2** (Er)	**3** (Er)	**4** (Er)	**5** (Er)	**6** (Y)
Charge of Er (Y) complex	0	0	0	-1	-1
Er-O1$_{equatorial}$	2.218(3)	2.244(2)	2.248(5)	2.246(3)	2.256(3)
Er-O2$_{equatorial}$	2.244(3)	2.242(2)	2.278(5)	2.246(3)	2.256(3)
Er-N2$_{equatorial}$	2.429(3)	2.418(2)	2.407(6)	2.425(4)	2.436(3)
Er-N4$_{equatorial}$	2.404(4)	2.401(2)	2.452(6)	2.425(4)	2.436(3)
Er-N3$_{equatorial}$	2.404(4)	2.431(2)	2.436(6)	2.424(6)	2.428(4)
Er-O$_{axial}$	2.275(3)	2.329(2)	2.316(5)	-	-
Er-O$_{axial}$	-	-	-	-	-
Er-N$_{axial}$	-	-	2.232(7)	-	-
Er-Cl$_{axial}$	2.609(1)	2.601(1)	-	2.586(2)	2.599(2)
Er-Cl$_{axial}$	-	-	-	2.654(2)	2.661(2)

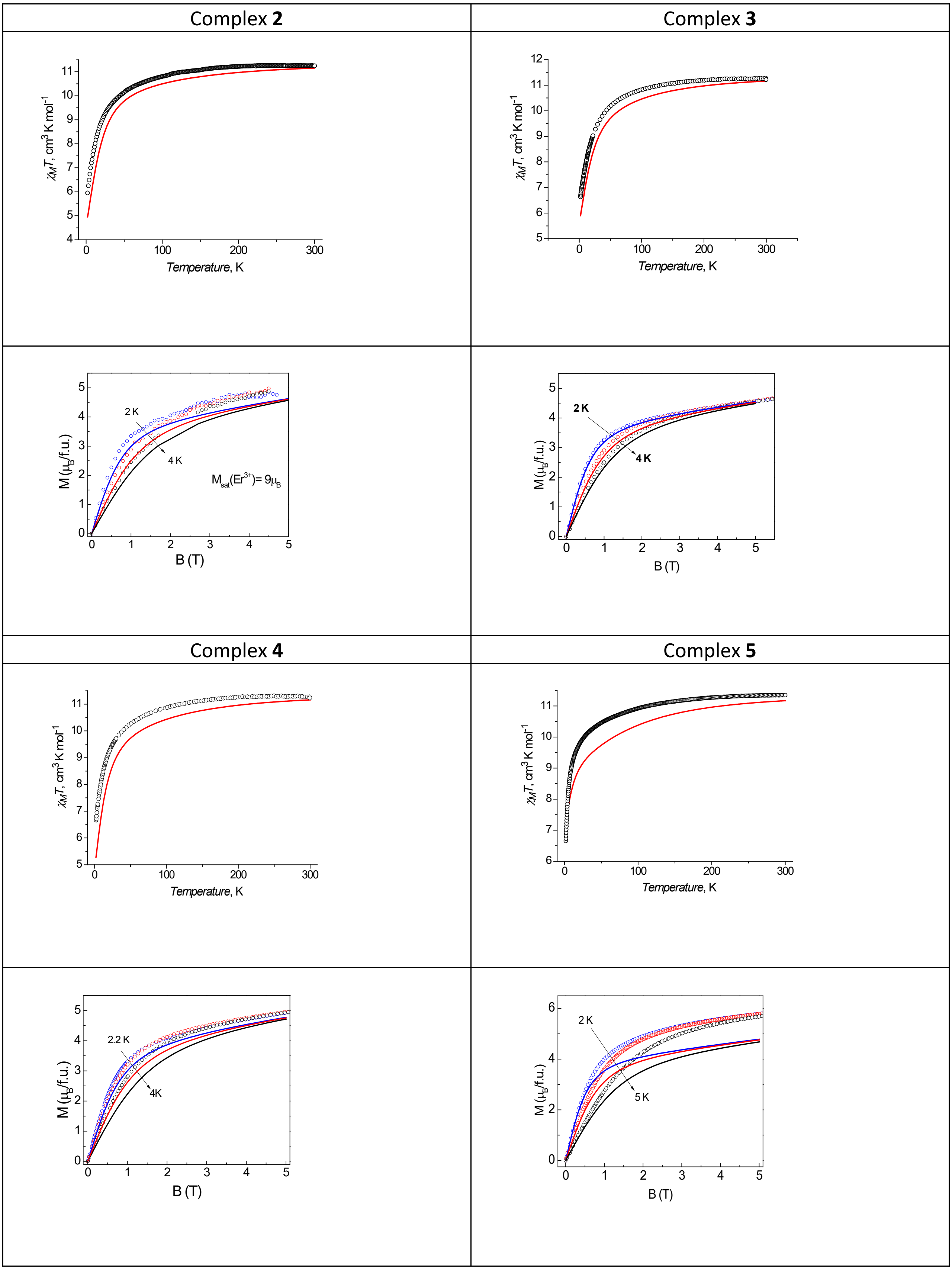

Figure S12. Experimental (open circles) and theoretical (solid lines) temperature dependences of magnetic susceptibility (in the form of χT *vs.* T) and field dependences of magnetization for complexes **2-5**. Theoretical data are obtained from *ab initio* calculations at the CASSCF/RASSI-SO/SINGLE_ANISO level of theory using the [Open]MOLCAS program.

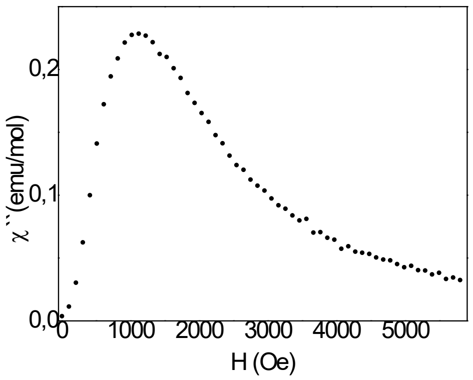

a

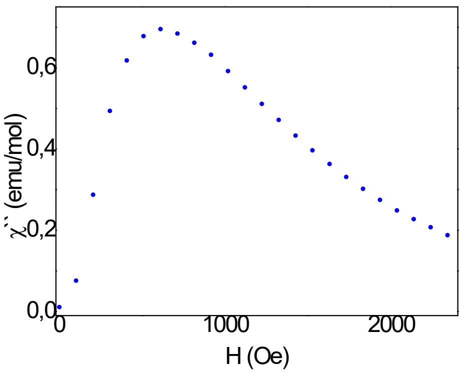

b

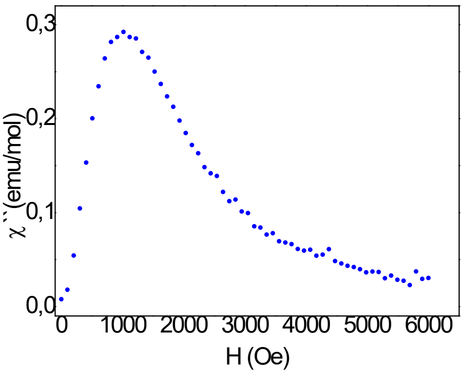

c

Figure S13. The field-dependence of the χ`` at T=2 K and fixed frequency ac excitation for complexes **2** (**a**, ν = 100 Hz), **3** (**b**, ν = 500 Hz) and **4** (**c**, ν = 100 Hz).

Table S10. Calculated intrinsic CF parameters b_4(L) and b_6(L) (where L=O, N, Cl) (in cm^{-1}) of the superposition CF model providing the optimum fit to the magnetic susceptibility of complexes **2-5** (Fig. 7)

**2**	**3**	**4**	**5**
b_4(O) = 518.5	b_4(O) = 559.3	b_4(O) = 312.1	b_4(O) = 300.5
b_6(O) = 176.2	b_6(O) = 271.9	b_6(O) = 319.0	b_6(O) = 279.5
b_4(N) = 374.7	b_4(N) = 277.2	b_4(N) = 285.0	b_4(N) = 262.9
b_6(N) = 267.0	b_6(N) = 219.3	b_6(N) = 163.2	b_6(N) = 161.0
b_4(Cl) = 318.1	b_4(Cl) = 446.2	$b_4(N_{azide})$ = 293.1	b_4(Cl) = 392.0
b_6(Cl) = 201.1	b_6(Cl) = 230.4	$b_6(N_{azide})$ = 203.9	b_6(Cl) = 199.1

Table S11. Calculated CF parameters B_{kq} (cm^{-1}) corresponding to the optimum fit to the magnetic susceptibility of complexes **2-5** (Fig. 7). Real (Re) and imaginary (Im) parts of the complex B_{kq} parameters are indicated

		**2**		**3**		**4**		**5**	
k	*q*	ReB_{kq}	ImB_{kq}	ReB_{kq}	ImB_{kq}	ReB_{kq}	ImB_{kq}	ReB_{kq}	ImB_{kq}
2	0	-138.6	0	20.4	0	239.6		118.6	0
2	1	-6.1	5.7	0.8	-1.1	-0.7	-7.7	0.0	0.1
2	2	28.2	4.5	-2.7	-0.4	1.4	-2.4	27.1	0.2
4	0	1589.2	0	1668.5	0	1053.0	0	1207.7	0
4	1	-8.4	49.6	-3.7	55.8	-72.0	-10.9	-0.1	38.4
4	2	-84.7	-44.8	-77.9	-45.8	-49.5	14.2	-83.6	-0.5
4	3	-6.7	-32.4	-6.0	-48.1	-4.3	-8.4	0.0	4.3
4	4	-396.8	43.4	-485.3	42.2	-203.7	41.7	-213.3	-2.7
6	0	-32.6	0	55.8	0	176.9	0	-10.2	0
6	1	-9.2	5.7	-17.2	-4.8	69.8	-25.0	0.0	10.1
6	2	28.9	8.9	31.2	8.2	24.2	-12.4	9.7	0.1
6	3	4.0	-3.0	4.1	20.7	15.6	39.8	0.0	-4.9
6	4	45.3	-19.9	129.6	-21.7	150.9	-11.6	156.2	2.0
6	5	8.7	69.5	7.9	69.9	-43.7	95.4	0.2	-13.8
6	6	-394.0	17.0	-362.0	7.50	-293.0	48.8	-284.0	-5.5
$S^{(a)}$, cm^{-1}		590.2		623.9		410.6		439.8	

[a]The *S* criterion estimates the overall strength of the crystal-field potential of Ln^{3+} ions in terms of B_{kq} parameters,

$$S = \left[\frac{1}{3} \sum_{k=2,4,6} \left(\frac{1}{2k+1} \right) \left(B_{k0}^2 + 2\sum_{q>0} (\mathrm{Re}\, B_{kq}^2 + \mathrm{Im}\, B_{kq}^2) \right) \right]^{1/2}$$

[Chang, N.C.; Gruber, J.B.; Leavitt, R.P.; Morrison, C.A. Optical spectra, energy levels, and crystal-field analysis of tripositive rare earth ions in Y_2O_3. I. Kramers ions in C_2 sites. *J. Chem. Phys.* **1982**, *78*, 3877-3889].

Table S12. Results of fit of $\chi''(\nu)$ and $\chi'(\nu)$ plots by generalized Debye model at 1120 Oe for **2**

T, K	α	τ, s	χ_{TS}, emu/mol	χ_S, emu/mol
2	0.35	9.3×10^{-5}	1.89	0.34
2.15	0.3	7.55×10^{-5}	1.81	0.37
2.3	0.25	5.8×10^{-5}	1.74	0.38
2.45	0.2	4.27×10^{-5}	1.67	0.39
2.6	0.16	3×10^{-5}	1.6	0.41
2.75	0.13	2.05×10^{-5}	1.55	0.42

Table S13. Results of fit of $\chi''(\nu)$ and $\chi'(\nu)$ plots by generalized Debye model at 610 Oe for **3**

T, K	α	τ, s	χ_S, emu/mol	χ_{TS}, emu/mol
2	0.2	1.81×10^{-4}	0.86	2.1
2.15	0.19	1.26×10^{-4}	0.83	1.99
2.3	0.18	8.93×10^{-5}	0.79	1.89
2.45	0.16	6.54×10^{-5}	0.77	1.79
2.6	0.15	4.9×10^{-5}	0.75	1.7
2.75	0.14	3.7×10^{-5}	0.73	1.61
2.9	0.12	2.8×10^{-5}	0.71	1.53
3.05	0.11	2.17×10^{-5}	0.7	1.45

Table S14. Results of fit of $\chi''(\nu)$ and $\chi'(\nu)$ plots by generalized Debye model at 1000 Oe for **4**

T, K	α	τ, s	χ_S, emu/mol	χ_{TS}, emu/mol
2	0.18	1.33×10^{-4}	0.38	2.08
2.15	0.2	1.05×10^{-4}	0.35	2.13
2.3	0.19	7.42×10^{-5}	0.34	2.07
2.45	0.17	4.95×10^{-5}	0.32	2.02
2.6	0.16	3.2×10^{-5}	0.31	1.97
2.75	0.16	1.91×10^{-5}	0.25	1.97

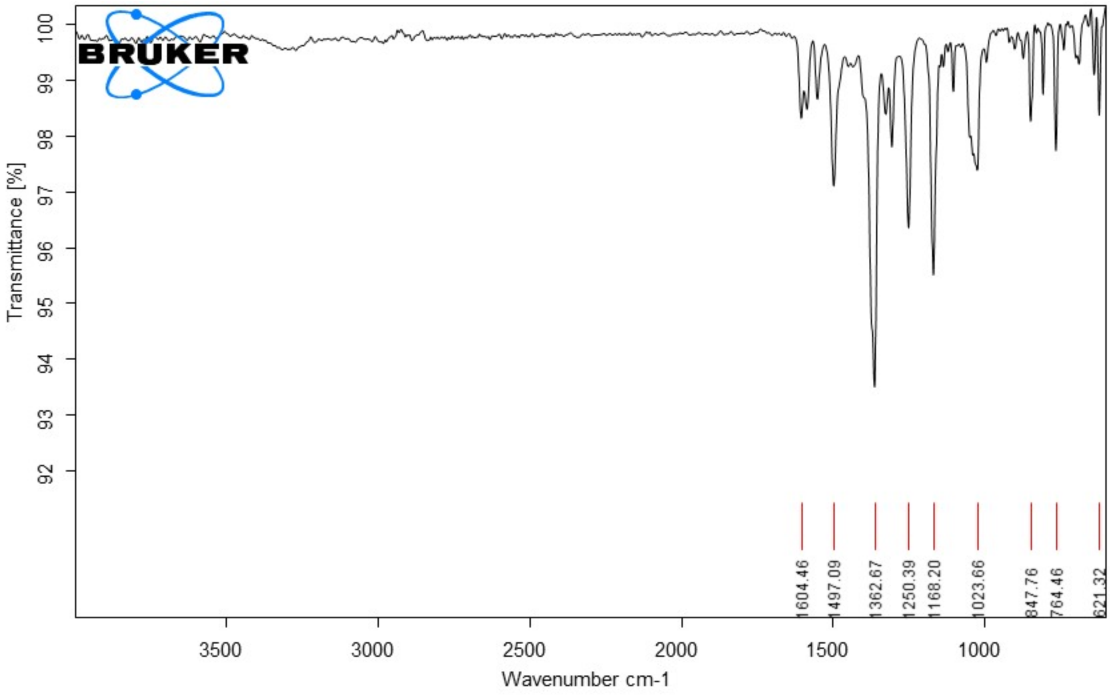

Figure S14. IR-spectrum of the complex **[Er(DAPMBH)(C_2H_5OH)Cl] (1)**

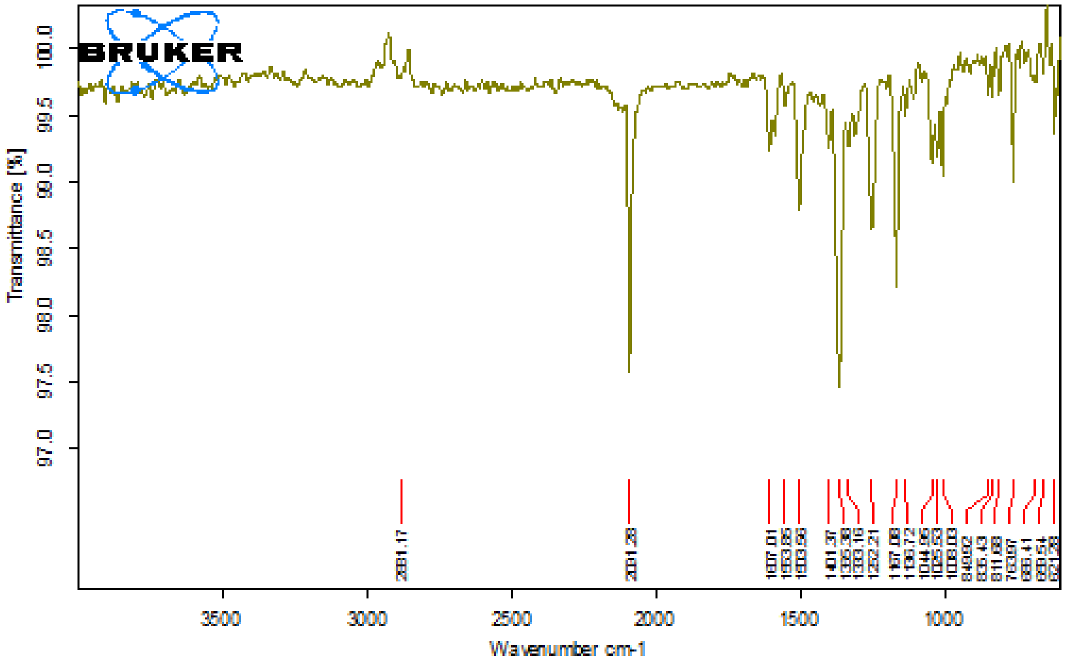

Figure S15. IR-spectrum of the complex **[Er(DAPMBH)(CH_3OH)N_3] (4)**

Table S15. Crystal data and structural refinement parameters for complexes **2-6**

	**2**	**3**	**4**	**5**	**6**
Chemical formula	$C_{29}H_{37}ClErN_5O_7$	$C_{26}H_{27}ClErN_5O_5$	$C_{26}H_{27}ErN_8O_5$	$C_{29}H_{35}Cl_2ErN_6O_4$	$C_{29}H_{35}Cl_2Er_{0.05}Y_{0.95}N_6O_4$
Formula weight	770.34	692.23	698.81	769.79	695.36
Cell setting	triclinic	triclinic	monoclinic	orthorhombic,	orthorhombic,
Space group, Z	$P\bar{1}$, 2	$P\bar{1}$, 2	$P2_1/c$, 4	$Cmc2_1$, 4	$Cmc2_1$, 4
λ (Å)	0.71073	0.71073	0.78790	0.71073	0.71073
Temperature (K)	120(1)	150(1)	100(2)	100(1)	150(1)
a (Å)	8.3532(2)	7.0338(1)	13.869(3)	18.7290(5)	18.7925(12)
b (Å)	13.8859(2)	11.4540(3)	10.631(2)	14.8702(4)	14.9064(8)
c (Å)	14.6874(4)	16.6083(4)	17.890(4)	11.1918(4)	11.1790(7)
α (°)	88.386(2)	90.897(2)	90	90	90
β (°)	80.033(2)	98.063(1)	97.234(3)	90	90
γ (°)	83.244(2)	104.417(2)	90	90	90
Cell volume ($Å^3$)	1666.20(7)	1281.32(5)	2616.7(10)	3117.0(2)	3131.6(3)
ρ (g/cm^3)	1.535	1.794	1.774	1.640	1.475
μ, cm^{-1}	26.48	34.27	42.70	29.08	21.18
Crystal size, mm^3	0.12×0.21×0.24	0.04×0.21×0.47	0.10×0.08×0.06	0.10×0.10×0.25	0.10×0.10×0.30
Refls collected/unique	18540 / 9018	27236 / 8819	17989 / 4628	5094 / 3427	4307 / 3277
R_{int}	0.0324	0.0269	0.0430	0.0327	0.0276
θ_{max} (°)	26.00	31.00	28.003	29.06	26.31
Parameters refined	484	351	370	212	212
Final R_1, wR_2 [I>2σ(I)]	0.0447, 0.1028	0.0241, 0.0521	0.0537, 0.1347	0.0331, 0.0533	0.0343, 0.0710
Goodness-of-fit	1.003	1.003	1.062	0.961	1.059
CCDC number	2107393	2107394	2100722	2099114	2100999
