# Supplementary material for: A Series of Novel Pentagonal-Bipyramidal Erbium(III) Complexes with Acyclic Chelating N3O2 Schiff-Base Ligands: Synthesis, Structure, and Magnetism
Source: Molecules. 2021 Nov 16;26(22):6908. doi: 10.3390/molecules26226908 (PMC8622354; doi:10.3390/molecules26226908)

checkCIF/PLATON report

Structure factors have been supplied for datablock(s) er2

THIS REPORT IS FOR GUIDANCE ONLY. IF USED AS PART OF A REVIEW PROCEDURE FOR PUBLICATION, IT SHOULD NOT REPLACE THE EXPERTISE OF AN EXPERIENCED CRYSTALLOGRAPHIC REFEREE.

No syntax errors found. CIF dictionary Interpreting this report

Datablock: er2

Bond precision: C-C = 0.0108 A Wavelength=0.78790

Cell:	a=13.869(3)	b=10.631(2)	c=17.890(4)
	alpha=90	beta=97.234(3)	gamma=90
Temperature:	100 K		

	Calculated	Reported
Volume	2616.7(10)	2616.7(10)
Space group	P 21/c	P 21/c
Hall group	-P 2ybc	-P 2ybc
Moiety formula	C26 H27 Er N8 O5	C26 H27 Er N8 O5
Sum formula	C26 H27 Er N8 O5	C26 H27 Er N8 O5
Mr	698.82	698.81
Dx,g cm-3	1.774	1.774
Z	4	4
Mu (mm-1)	4.242	4.270
F000	1388.0	1388.0
F000'	1387.47	
h,k,lmax	16,12,21	16,12,21
Nref	4644	4628
Tmin,Tmax	0.697,0.774	0.001,1.000
Tmin'	0.621	

Correction method= # Reported T Limits: Tmin=0.001 Tmax=1.000
AbsCorr = EMPIRICAL

Data completeness= 0.997 Theta(max)= 28.003

R(reflections)= 0.0537(3912) wR2(reflections)= 0.1417(4628)

S = 1.062 Npar= 370

The following ALERTS were generated. Each ALERT has the format
test-name_ALERT_alert-type_alert-level.
Click on the hyperlinks for more details of the test.

Alert level A

```
PLAT972_ALERT_2_A Check Calcd Resid. Dens.  0.72A   From Er1            -4.45 eA-3
```

Author Response: In our opinion, the unefficient absorption correction (rather usual at synchrotron radiation wavelength ca. 0.8 A) are responsible of the residual densities on heavy atom.

Alert level C

```
PLAT342_ALERT_3_C Low Bond Precision on  C-C Bonds ...............    0.01077 Ang.
PLAT906_ALERT_3_C Large K Value in the Analysis of Variance ......      2.430 Check
PLAT911_ALERT_3_C Missing FCF Refl Between Thmin & STh/L=    0.596         17 Report
PLAT971_ALERT_2_C Check Calcd Resid. Dens.  0.85A   From Er1             2.37 eA-3
PLAT971_ALERT_2_C Check Calcd Resid. Dens.  0.93A   From Er1             2.19 eA-3
PLAT971_ALERT_2_C Check Calcd Resid. Dens.  0.89A   From Er1             2.07 eA-3
PLAT971_ALERT_2_C Check Calcd Resid. Dens.  1.05A   From O1              1.62 eA-3
PLAT975_ALERT_2_C Check Calcd Resid. Dens.  0.85A   From O1              0.67 eA-3
PLAT975_ALERT_2_C Check Calcd Resid. Dens.  0.81A   From N6              0.48 eA-3
PLAT976_ALERT_2_C Check Calcd Resid. Dens.  0.85A   From O2             -0.50 eA-3
```

Alert level G

```
ABSMU01_ALERT_1_G  Calculation of _exptl_absorpt_correction_mu
                  not performed for this radiation type.
PLAT083_ALERT_2_G SHELXL Second Parameter in WGHT  Unusually Large      20.20 Why ?
PLAT092_ALERT_4_G Check: Wavelength Given is not Cu,Ga,Mo,Ag,In Ka    0.78790 Ang.
PLAT909_ALERT_3_G Percentage of I>2sig(I) Data at Theta(Max) Still       72% Note
PLAT913_ALERT_3_G Missing # of Very Strong Reflections in FCF ....         3 Note
PLAT933_ALERT_2_G Number of OMIT Records in Embedded .res File ...        12 Note
PLAT941_ALERT_3_G Average HKL Measurement Multiplicity ..........        3.9 Low
PLAT978_ALERT_2_G Number C-C Bonds with Positive Residual Density.         1 Info
```

1 **ALERT level A** = Most likely a serious problem - resolve or explain
0 **ALERT level B** = A potentially serious problem, consider carefully
10 **ALERT level C** = Check. Ensure it is not caused by an omission or oversight
8 **ALERT level G** = General information/check it is not something unexpected

1 ALERT type 1 CIF construction/syntax error, inconsistent or missing data
11 ALERT type 2 Indicator that the structure model may be wrong or deficient
6 ALERT type 3 Indicator that the structure quality may be low
1 ALERT type 4 Improvement, methodology, query or suggestion
0 ALERT type 5 Informative message, check

It is advisable to attempt to resolve as many as possible of the alerts in all categories. Often the minor alerts point to easily fixed oversights, errors and omissions in your CIF or refinement strategy, so attention to these fine details can be worthwhile. In order to resolve some of the more serious problems it may be necessary to carry out additional measurements or structure refinements. However, the purpose of your study may justify the reported deviations and the more serious of these should normally be commented upon in the discussion or experimental section of a paper or in the "special_details" fields of the CIF. checkCIF was carefully designed to identify outliers and unusual parameters, but every test has its limitations and alerts that are not important in a particular case may appear. Conversely, the absence of alerts does not guarantee there are no aspects of the results needing attention. It is up to the individual to critically assess their own results and, if necessary, seek expert advice.

Publication of your CIF in IUCr journals

A basic structural check has been run on your CIF. These basic checks will be run on all CIFs submitted for publication in IUCr journals (*Acta Crystallographica*, *Journal of Applied Crystallography*, *Journal of Synchrotron Radiation*); however, if you intend to submit to *Acta Crystallographica Section C* or *E* or *IUCrData*, you should make sure that full publication checks are run on the final version of your CIF prior to submission.

Publication of your CIF in other journals

Please refer to the *Notes for Authors* of the relevant journal for any special instructions relating to CIF submission.

PLATON version of 13/07/2021; check.def file version of 13/07/2021

Datablock er2 - ellipsoid plot

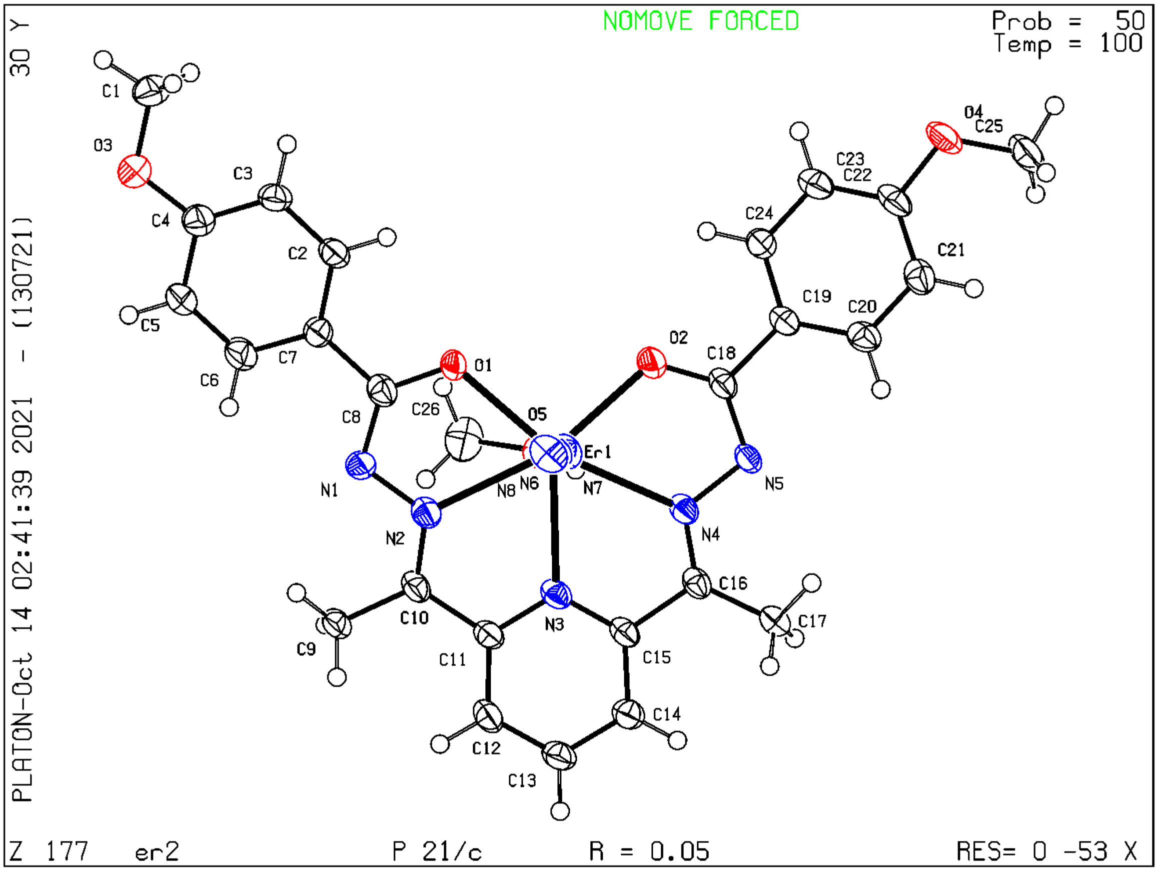

Supplement: Supplementary file 1 [file 4_checkcif.pdf › Supplementary files/4_checkcif.pdf]
